# Acute hypoxia alters visuospatial attention orienting: an electrical neuroimaging study

**DOI:** 10.1038/s41598-023-49431-4

**Published:** 2023-12-20

**Authors:** A. Zani, N. Crotti, M. Marzorati, A. Senerchia, A. M. Proverbio

**Affiliations:** 1 School of Psychology, Vita-Salute San Raffaele University, Via Olgettina 58-60, 20132 Milan, MI Italy; 2 Department of Psychology, University of Milan-Bicocca, Milan (MI), Italy; 3 Institute of Biomedical Technologies, National Research Council (CNR ITB), Segrate, MI Italy

**Keywords:** Neuroscience, Psychology

## Abstract

Our study investigated the effects of hypoxia on visuospatial attention processing during preparation for a single/double-choice motor response. ERPs were recorded in two sessions in which participants breathed either ambient-air or oxygen-impoverished air. During each session, participants performed four cue-target attention orienting and/or alerting tasks. Replicating the classic findings of valid visuospatial attentional orienting modulation, ERPs to pre-target cues elicited both an *Anterior directing attention negativity* (ADAN)/CNV and a posterior *Late directing attention positivity* (LDAP)/TP, which in ambient air were larger for attention orienting than for alerting. Hypoxia increased the amplitude of both these potentials in the spatial orienting conditions for the upper visual hemifield, while, for the lower hemifield, it increased ADAN/CNV, but decreased LDAP/TP for the same attention conditions. To these ERP changes corresponded compensatory enhanced activation of right anterior cingulate cortex, left superior parietal lobule and frontal gyrus, as well as detrimental effects of hypoxia on behavioral overt performance. Together, these findings reveal for the first time, to our knowledge, that (1) these reversed alterations of the activation patterns during the time between cue and target occur at a larger extent in hypoxia than in air, and (2) acute normobaric hypoxia alters visuospatial attention orienting shifting in space.

## Introduction

Neuroscientific and physiological literature have provided evidence that hypoxia affects cognitive and physiological processes of the brain, depending on severity, duration of exposure and individual susceptibility (e.g., Refs.^1–4^). For example, acute exposure to high altitude-related hypobaric hypoxia can induce neurological changes of various types^5^, as cerebral hypoxia, which is associated to cognitive fatigue and a reduction of cognitive performance. Ochi et al.^6^ discovered that 10 min of moderate-intensity exercise under hypoxic conditions have a negative impact on executive performance and neural activity in the left dorsolateral prefrontal cortex. Environmental hypoxia alters posterior cingulate cortex metabolism during memory tasks^7^ and may even alter the function of serotonin ^1A^receptor, involved in cognition and memory^8^. Chronic exposure to normobaric hypoxia increases testosterone levels and testosterone/cortisol ratio in cyclists^9^. Other studies have shown how hypoxia is detrimental to various mental processes and cognitive functions^10^, including working memory^11^, vigilance (e.g., Ref.^12^), and motor reactivity (e.g., Ref.^13^).

However, large methodological differences across studies, including disparity in altitude, duration of the hypoxic exposure (namely, acute, short-term, long-term or chronic hypoxia), the different methods by which acute hypoxia was obtained (normobaric or hypobaric), the subjects variability, the task used, and the different Event-related potential (ERP) components considered, make drawing exhaustive conclusions about the deleterious effects of hypoxia on neural processes subserving cognition and spatial attention rather problematic. Importantly, this also arises from the insufficiency of ERPs intracerebral localization source analyses by the whole of the studies in the literature.

As for the effects of acute hypoxia on electrophysiological markers of attention allocation and target selection processing, it has been shown that this condition noticeably increased EEG alpha rhythm (e.g., Refs.^14,15^), or reduced the amplitude of several scalp-recorded ERP components. At this regard, while investigating the effects of acute hypoxia on visual sustained attention, Altbäcker et al.^16^ found that whereas both a task-relevant target-related Go P300 and a target-irrelevant NoGo P300 efficiency remained unchanged, a novelty P3a decreased significantly_._ Furthermore, a more recent investigation on the effects of hypoxia on attention, as reflected by so called visual mismatch negativity (vMMN), demonstrated a significant 50% reduction in the amplitude of this component during hypoxia^17^.

Most importantly, it has been demonstrated that hypoxia significantly affects late latency components, including P300 and Contingent Negative Variation (CNV). For example, both behavioral performance and P300 latency were reported to be significantly delayed, by hypoxic conditions in oddball paradigms^18–20^. Interestingly, Hayashi et al.^18^ showed how hypoxia affected P300 latency more than hypobaric altitude, since administration of oxygen countered the effects of simulated hypobaric conditions. Most intriguingly, Kida and Imai^19^ found that the P3 component was followed by a negative on-going (frontal maximum) and positive on-going (parietal maximum) slow waves”; both the CNV-like frontal negative slow wave and the positive parietal slow wave progressively increased in amplitude as hypobaric hypoxia increased. Interestingly, although these same slow-waves were found in the ERPs of participants who demonstrated delayed RTs at high altitudes, when the participants failed in the go/no-go RTs task, both slow-waves either disappeared or diminished, thus suggesting that they might be associated with attempts to maintain behavioral responses against the deteriorative workload effects of hypoxia. Still, a later study by Takagi and Watanabe^21^ on the acute effects of hypobaric hypoxic conditions found that the amplitude of a late CNV (l-CNV) showed a negative correlation with RTs at the altitudes of 3000 and 0 m. Conversely, at 4000 m and above this negative correlation concerned the amplitude of an early CNV (e-CNV), despite at 6000 m the complete slow-wave amplitude decreased extensively compared with that at sea level (or 0 m).

Overall, the electrophysiological results reviewed above indicated that acute hypoxia (either normobaric or hypobaric) deleteriously affected neural processing of relevant stimuli falling at an attended space location and psychomotor performance in an altitude- and time-dependent way. It is peculiar, in our view that no study, to our knowledge, has considered whether hypoxia affects the functional activation of the neural network undergirding the shifting of visual attention in space induced by pre-cueing modes, as reflected by ERPs rising in the processing profile between the cue and target presentation. This, probably, in line with the limited number of studies in the attention literature which coped with the time course of hypoxic modulation of neural activations elicited by the presentation of cue stimuli preceding targets in typical attentional cue-target paradigms as those devised by Michael Posner (e.g., Refs.^22,23^).

Even if different variants of these paradigms exist, ordinarily they include the presentation of an attentional cue and later, after a certain delay, the delivery of a target stimulus to which a behavioral response must be given, at either the cued space location (i.e., cued, or valid targets) or an uncued location (i.e., uncued or invalid targets.). Whatever neurocognitive response or performance benefits for cued targets to a point in space are thought to be dependent on cue capacity to direct attention to that point, while the timing of attentional orienting is indicated by the variations in the processing profile across the cue-target time span^24^. Harter and Guido^25^ and Harter et al.^26^ who were among the first at investigating the neural mechanisms of shifting attention in the visual space introduced a new method for computing arbitrary difference ERP (or DERP) waves between the left- and the right-orienting conditions in a Posner endogenous cueing lateralized task. Thanks to the computations of these DERP subtractions and their analysis in the cue-target time span, three different ERP components, contralateral to the direction of attention shift, were described, and further investigated:

the EDAN (*Early directing attention negativity*) defined as an early perceptual negative wave (beginning about 200 ms after cue) mostly on right occipital-parietal posterior electrodes;

the ADAN (*Anterior directing attention negativity*), described as an increased slow wave negativity centered at frontal electrodes with a typical time window around 310–440 ms post-cue^27^; and,

the LDAP (Late directing attention positivity) defined as a late-latency positivity (starting ~ 400 ms after cue) developing at posterior electrodes contralateral to the direction of attentional shifting cueing (e.g., Refs.^28–30^).

Comparing the attentional modulation induced by central and peripheral cues, Yamaguchi et al.^28^ reported that central cues elicited an enhanced *Contingent Negative Variation* (CNV), (a negative shift like Harter’s EDAN) starting about 240 ms after cue presentation over posterior scalp sites contralateral to the cued visual field. Other authors have found that “attending” and “interpreting” a centrally presented cue was associated with elicited similar sensory processing activity in the first 350 ms at occipital extrastriate areas. This initial activation was followed by a long-lasting contralateral negative variation (preceding the target occurrence) *biasing-related negativity (BRN)*—at frontoparietal areas^31,32^. Conversely, in Yamaguchi’s et al.^28^ study, peripheral cues mostly enhanced an N1 component (i.e., 140–200 ms post cue) over the contralateral hemisphere. Following N1 enhancement, a sustained negative potential shift appeared after 400 ms post cue at posterior scalp sites contralateral to cued visual field. The results by further studies reinforce the notion that early response preparation—or biasing—processes of the posterior-parietal cortex are triggered by the spatial cueing stimuli, perhaps in an initial step of attentional orienting, together with a lateral-prefrontal involvement, possibly related to the voluntary control and maintenance of attentional shift^25–30,33,34^.

No matter the cueing location, the sustained negative potentials found to be developing across the cue-target time span while participants’ attention is directed to a point in space or to planning an action in relation to a sequence of stimuli—such as a decision process, a motor action, or inhibition of a motor action—may be considered an expectation negativity, which is like the so-called contingent negative variation (CNV) potential. It is theorized that this deflection is the expression of suppression processes of ongoing activity for preparation and orientation during the time between warning and target in view of performing a required rapid response to the task^22,23,35–38^. fMRI indexes indicated that this negative shift during the warning interval appears to arise in the anterior cingulate cortex (ACC) and adjacent structures^39^, which hints at its overlapping with the executive network^40^.

As for the modulation of ERPs CNV-like component (or ADAN) by phasic attention alerting and/or orienting, a few studies reported controversial findings by using drastically modified versions of Fan’s et al.^41^ *Attention Networks Test* (ANT) for investigating vigilance. For instance, using a short 600 ms cue-target ISI and an alerting auditory tone cue, Abundis-Gutierrez et al.^42^ found that while phasic alerting induced a significant CNV modulation at anterior scalp sites, attention orienting did not. A result, among several others, that was most recently replicated by Luna et al.^43^ using a different version of ANT, but similar short ISI and auditory tone, from that used by Abundis-Gutierrez et al.^42^. However, these findings are opposed to those indicated by Galvao-Carmona et al.^44^ who, using a different ANT version with a lengthened cue-target ISI of 1 s, reported that CNV amplitude at anterior scalp increased as a function of information provided by the cue, so to have, rather, a larger amplitude for attention orienting, and, in turn, a lower amplitude for attention alerting, it being CNV larger to CC than NC.

These controversial findings could be thought to result from the shift from an initial exogenous (or reflexive) shift of attention due to a short cue-target ISI, as in Ref.^42^^,^^43^, to an endogenous (or voluntary) modality of attentional orientation, as in Ref.^44^, due to the prolonged cue-target ISI of 1 s. Possibly, in fact, in line with Corbetta’s and Shulman’s^45^ two-systems attention model, the indicated reflexive mode of attention orienting might also be the reason why Yamaguchi et al.^28^ did not find any significant contralateral attention orienting effects at anterior scalp sites for none of the cue-target ISI spans (200, 500, and 800 ms) in the peripheral cueing experiment as compared to the central cueing one.

In the light of all the matters reviewed above, we carried out the present study with manifold aims. Our primary goal was to examine the functional activation of attention alerting and/or orienting neural networks, and the potential influence of respiratory hypoxia, as indicated by ADAN/CNV and LDAP/TP slow components, on these networks. In addition, we aimed to identify any differences in the activation and impact trends as a result of varying levels of cognitive and motor workload. Furthermore, the aim of this investigation was to examine potential hemispheric asymmetries in the functional trends of these components. To achieve this, we employed the modified Attention Network Test (ANT)^15^ which incorporates the three ANT initial central cue (CC), no cue (NC), and local cue (LC) cueing modes^41^, as well as a motor local cue (LCmot) cueing modality (Ref.^15^ as outlined in the Procedure).

Second, we wished to investigate whether the above ERP components and the separate attentional networks from which they are undergirded showed a different modulation as related to the above and below horizontal meridian (HM) of the visual field, to further investigate the presence of possible spatial anisotropy (e.g., Refs.^46–48^ in favour of the upper hemifield.

Importantly, we also aimed to assess whether close, but separate, interrelationships could be evidenced in the activation trends shown by the anterior and posterior brain regions, respectively. This because, in line with current single-units activation-based “*brain interconnectivity theory*” recently advanced by Snyder et al*.*^49^, a division of labor would be carried out across cortical districts. According to this theory, while anterior executive control areas would induce the shifting of attention in space and its sustaining over time, visual cortical areas would be closely related to a corresponding biased preparation for later target processing.

Most importantly, we aimed to assess whether hypoxia affected both cue-dependent attention alerting and attention shifting of visuospatial attention networks or, separately, only one of these systems, and which brain areas involved in shifting the attentional focus in space were affected by acute normobaric hypoxia by means of intracranial source analyses.

To these aims, a demanding but not harmful bout of respiratory hypoxia was induced in a sample of voluntary participants so to be possibly able to measure its effects on attentional control of visuospatial attention shifting in space.

## Methods

### Participants

Ten healthy volunteers (4 females and 6 males) aged 19 to 27 years (M = 24, SE = 2.7), participated in the current experimental research. Participants were excluded if they had a history of neurological and psychological syndromes or had taken any psychopharmacological substances. Additionally, individuals with a history of cigarette smoking, arterial hypertension, and cardiovascular or respiratory diseases were also excluded from the study. Furthermore, to demote confounding effects, no participant had to have sojourned at a higher altitude than 300–400 m in the 4 weeks preceding the study nor had to have been regularly and intensively engaged in any physical training program. Again, participants were requested to refrain from any vigorous physical activity and from alcohol and caffeine- and theophylline-containing beverages in the 24 h prior to the recording sessions of the study. All participants selected for the study had normal or corrected-to-normal vision, with right-eye and right-hand dominance, and no left-handed relatives, as determined by the Edinburgh Inventory. Notwithstanding the completion of the two recording sessions of the study, two volunteers were later excluded from the following analyses due to intolerable eye- and body-movement artifacts rejection rates, making their signal-to-noise ratio unacceptable.

As with our previous studies (e.g. Refs.^15,47,48^, the current experimental method received approval from the ethics committee of the Italian National Research Council (CNR) and was conducted according to APA ethical standards (APA, Monitor Staff, 2003, vol. 34, n. 1) for the treatment of human participants. The recordings were also conducted with the understanding and the written consent of each participant in compliance with the indications of the 2018 Declaration of Helsinki ethical principles for medical research involving human subjects by the World Medical Association (WMA Declaration of Helsinki, 9 July 2018, PDF file).

### Post-hoc power analysis

To assess the statistical power of the subjects’ sample for this study, a *post-hoc* power analysis was conducted using G*Power 3.1.9.6^50^. The analysis revealed that the statistical power for the ERPs data was 1-β = 0.95 [7 participants, α = 0.05, Ƞ^2^ = 0.33 (*f* = 0.70)].

### Stimulus materials

**Figure 1:**
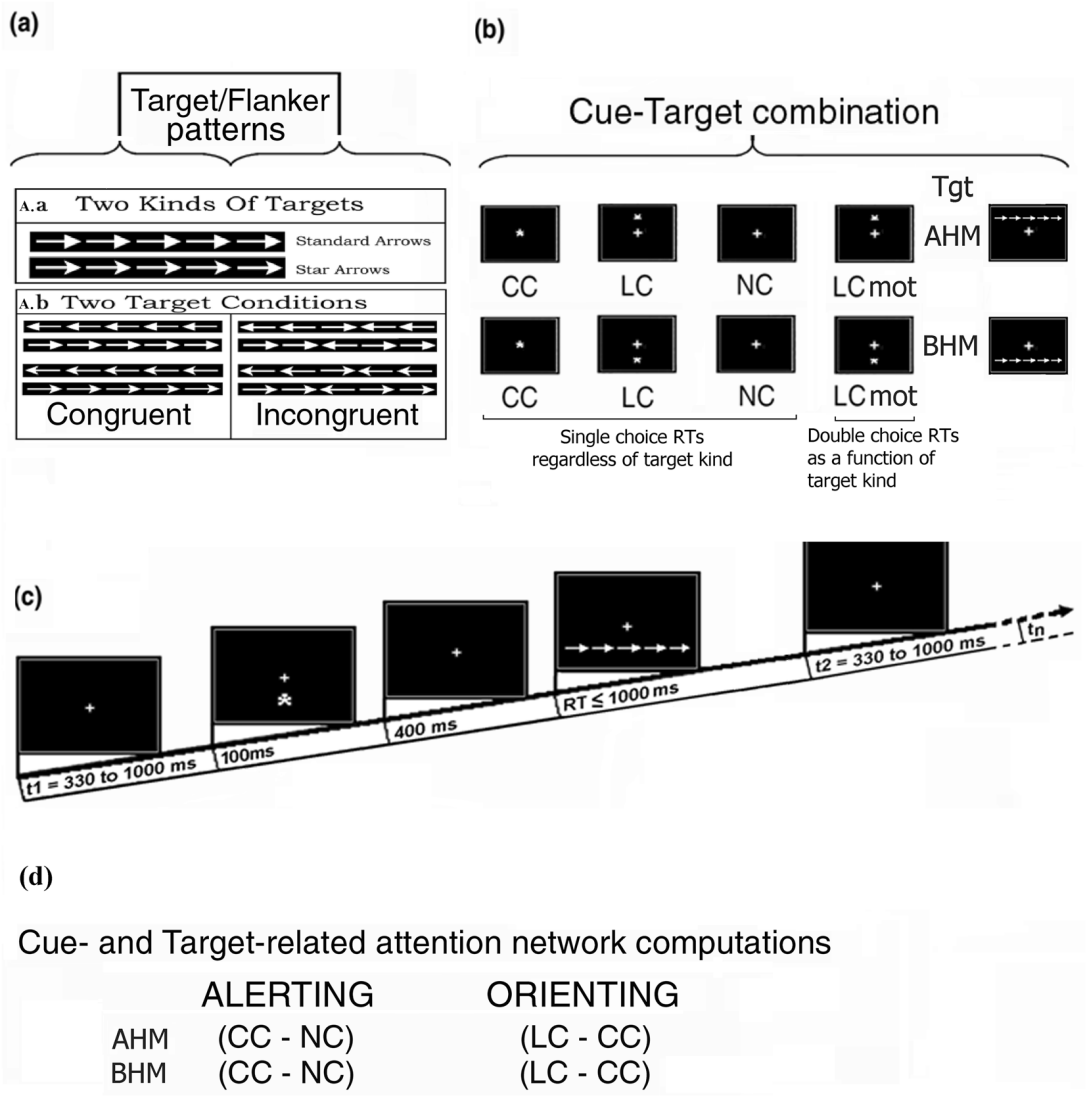
Graphical illustration of the characteristics of the modified Attention Network Test (ANT). In addition, (**b**) illustrates the stimulus materials and the different cue-target conditions used in the study. (**(a**); A. a): Two kinds of white arrow-target strings were used: the so-called “standard” and “star” arrows. The central arrow of the strings consisted of the true target while the flanking arrows on each side posed as potential distracters. ((**a**); A. b): Congruent and incongruent central targets Vs. flanker strings were presented and based on the direction of the arrow tip (left or right), eight (8) different target-flanker conditions resulted. (**b**): Four different cue-target and motor-task conditions were administered in randomized order: a *central cue* (CC) condition in which a cue overimposed on the central FC of the visual screen was later on followed by a vertically eccentric target with respect to the FC in either the above horizontal meridian (Above-HM) or the below horizontal meridian (Below-HM) visual monitor hemifield; a *local spatial cue* (LC or SC) in which a vertically eccentric cue with respect to the central FC was validly followed at the same point in space in either the Above-HM or Below-HM visual monitor hemifield by a target; a *no cue* (NC) condition in which the vertically eccentric targets simply followed one another appearing at random in the two visual monitor hemifields without being preceded by any priming cues. In these three conditions, the participants’ motor response to targets depended on a single-choice RTs button-press with the index finger of the hand on the same side as the central-arrow-direction, independent of the target-arrow type. In the fourth cuing condition, *LCmot*, volunteers had to discriminate both the target-arrow type and pointing direction to make a double-choice RTs button-press with the index or middle fingers of the hand on the same side as the arrow pointing direction. (**c**) Schematic representation of the time course of cue-target stimulus events administered on each trial of the study. (**d**) Computational procedures followed for analyzing the relevant attentional networks investigated. (Taken and modified from Posner et al.^22^).

Stimulus materials consisted of strings of five (5) contiguous arrows, serving as targets. Arrows were of two types: so called “standard” and “star” arrows (see Fig. 1a). While the former type had a tip with a vertically linear rear side, the latter showed a slightly inward-bound and oblique rear side nearby the arrow shaft. The central arrow of each string consisted of a true target while the flanking two arrows on each side of the latter posed as potential distracters. Overall, each target-and-flankers-string subtended 8.7 degrees of visual angle (Width) along the horizontal meridian and 1.3 degrees (Height at the arrow tip backside) along the vertical meridian (see Fig. 1A.a; These materials were the same as those used in Ref.^15^.

No matter the arrow types, the tip of the central target arrow could point to the left or to the right side, whereas the flanking arrows could point toward the same (Congruent flankers) or the opposite direction (Incongruent flankers) as the target. Overall, there were eight different target string combinations (see Fig. 1A.b). Besides arrow-strings, stimulus materials also consisted of asterisks, serving as cues for four different cue-target conditions. Both the asterisks and the arrow-strings were of white color. All these stimulus materials were presented on the blackened background of a 17 “cathode ray tube (CRT) screen in front of the volunteers.

The luminance of the asterisk cue amounted to 7.3 cd/m^2^. Conversely, the luminance assessments for the standard-arrow- and star-arrow-strings were 27.81 and 26.96 cd/m^2^, respectively, and were matched across arrow-pointing directions and target-flankers congruency.

### Procedure

The present study was conducted at the *Cognitive Electrofunctional Imaging Lab* of the Institute of Molecular Bioimaging and Physiology, National Research Council, Milan, Italy. As for the stimulus materials, the followed procedure was like that used in Ref.^15^. Participants took part in randomized order in two ~ 4 h (4 h) lasting experimental sessions, one week apart from each other. During these two experimental sessions they breathed either ambient-air (normoxia) or a 12.5% O^2^-impoverished air mixture (simulating respiration conditions at a high altitude of ~ 4200 m or ~ 13.780 feet—at sea level, which may be assimilated to an acute bout of pressing normobaric hypoxia) while performing in four different cue-target visuospatial attention conditions readapted starting from Fan’s et al. (2002) *Attention Network Test* (ANT)^41^.

As for the hypoxia session, just starting from their arrival to the laboratory the volunteers breathed a normobaric hypoxic mixture (~ 12.5% O^2^ in air, simulating an altitude of about 4200 m) obtained removing a controlled amount of oxygen from air by means of a MAG-10 apparatus (Higher Peak LLC, Winchester, MA, USA). The mixture was delivered through a facemask at 30 l min^-1^. Excess air flow was diverted outside the mask to prevent inspired oxygen pressure from increasing above 90 Torr.

Based on data from the physiological literature indicating that in humans the most relevant cardiorespiratory and blood plasmatic effects occur between 2 and 4 h of severe hypoxic breathing (see, e.g., Ref.^51^, all our volunteers were systematically subjected to EEG recording after 2 h of such a breathing condition, during which a 128-electrode electrocap was applied to their head. During this time span, they also completed paper and pencil mood and attention scales and performed an attention task based on the selective cancellation of alphabetic letters. As in Ref.^15^, for experimental standardization and comparison purposes, the subjects also started their task-related EEG recordings after 2 h during the normoxic session, a span of time during which they were engaged in the above-mentioned activities in addition to the electrocap application.

At the end of the two hours of preparation, the participants were made to sit in a comfortable easychair with a high backrest within an electrically and magnetically shielded cubicle (Faraday cage) in front of a CRT screen with a small white fixation cross (FC) in the center of its black background placed at 114 cm (or 3.34 feet) from them.

For each participant, during the whole duration of the procedures relative to the hypoxic session a physician was present for monitoring her/his physiological status and stopping the session at any moment upon any sign of sickliness. Participants were also informed to take off the breathing mask and stop the experimental session in case they became aware of any physical and neurocognitive unbearable sensations. Luckily enough, none of them felt to comply with these instructions.

During the EEG recordings, sequences of the white arrow-strings were randomly presented above or below the fixation-cross at the center of the visual screen. The central target-arrow of each string fell just in correspondence of the fixation cross with a vertical eccentricity of ± 1.25 degrees of visual angle from the latter. The arrow-strings were preceded or less by an asterisk-cue. Whenever presented, the cue fell at different vertically organized locations of the visual field so to build up different cueing modes. In fact, depending on the cue presentation locations (above or below the FC or centered over it) as well as onto target-related motor tasks to be performed, four (4) different alerting or valid spatial attention orienting sets were induced in the participants (Fig. 1b). More in details, the latter had to deal with: (1) a condition where cues were presented overimposed on the FC aimed at eliciting a phasic alert response followed later on by a target-related exogenous spatial orienting of attention to the point in space where the target-string was delivered (Central cue, CC); (2) a vertically eccentric (above or below the FC) cueing condition aimed at eliciting both a cue-related phasic alerting and an exogenous spatial orienting of attention (Local or Spatial cue, LC) to the point in space where the target string would have later on been validly delivered; and (3) the sequential presentation of vertically eccentric (above or below the FC) target strings without any priming cue, aimed at eliciting a possibly tonic and unspecific alerting or sustained attention condition over time (No Cue, NC). For each of these three cue conditions, on each trial the participants had to discriminate the direction towards which the central target-arrow-tip of the five-arrows-string pointed, independent of the arrow type presented (i.e., standard or star arrow) and the direction of the flanking-arrows (Congruent and Incongruent), and to perform a single-choice-RTs button-press with the index finger of the corresponding hand (right or left). Participants had also to deal with a fourth spatially informative cueing condition, like LC, with the difference that they had to perform a double-choice-RTs button-press based on the target-arrow type (Double-choice-RTs LC, that is LCmot) presented. Indeed, in the LCmot condition participants had to discriminate the type of target-arrows (i.e., standard- or star-target-arrow) besides the direction to which the latter were pointing to, so to perform a double-choice button-press with the index or middle fingers of the corresponding hand (right or left), respectively (see Fig. 1b; see also Ref.^15^ for this type of task).

To acquaint the participants with the task designated for each cue-target condition, volunteers read thorough written instructions and conducted a trial run involving 30 stimulus pairs prior to commencing the recording.

Following Zani et al.^15^, for each cueing condition there were four separate blocks of trials, each containing 128 trials grouped in a differently randomized order and lasting approximately 3.5 min. To avoid any confounding and systematic interacting effects of practice, fatigue, and hypoxia, we also randomized the order of administration of the respiratory conditions, of the cue-target conditions, of the type of target-arrows, of the falling of the target in the upper or lower visual field, and of blocks presentation across participants. On each trial, a cue stimulus appeared for 100 ms, followed 400 ms later by the presentation of an arrows-string, which remained on the screen for 1000 ms before its offset to avoid any possibly baffling target-offset, besides target-onset, related ERP recordings. Inter-trial interval (ITI) randomly varied in duration between 350 and 1000 ms (Fig. 1c).

The ASA Lab (Advanced Source Analysis Lab) software package (Ver. 4.1.0.4) from Advanced Neuro-Technologies Inc.^©^ (A.N.T. Inc., Enschede, The Netherlands), running on a local network consisting of two tabletop personal computers (PCs), was used for stimulus presentation and electrophysiological data recording, as well as for offline EEG processing (i.e., EEG selective averaging and grand-averaging for each condition) and ERPs automatic measurement, mapping, and intracranial source reconstruction.

After instructing participants to stare at the central FC of the CRT screen, not to move, and to avoid horizontal eye movements and blinks, we recorded electroencephalographic (EEG) and electrooculographic (EOG) signals in continuous mode during each run.

As mentioned above, the total duration of both breathing sessions, including experimental and rest periods, was ~ 4 h, of which the participants spent the first 2 h undergoing preparation for ERP recordings, psychometric testing, and instructional procedures while breathing low-oxygen air.

### EEG recording and analysis

In line with Ref.^15^ recording procedures, EEG was recorded from scalp electrodes mounted in an ANT elastic Waveguard 128-electrode electrocap. The electrodes were densely spaced all over the frontal, central, temporal, parietal, and occipital scalp-sites as proposed by the 5% system^52^ devised for high-spatial resolution EEG/ERP recordings. Two electrodes placed below and above the right eye recorded vertical eye movements, whereas two further electrodes placed at the outer canthi of the eyes recorded horizontal eye movements. Linked ears served as the reference lead, whereas a frontal electrode served as a ground site. Electrode impedance was below 5 KΩ. Both EEG and EOG continuous signals were acquired using directional-current (DC) amplifiers and a digitization rate of 512 samples/sec.

In agreement with the procedures followed by Ref.^15^, offline, automated rejection of electrical artifacts was performed before EEG averaging to discard epochs in which eye movements, blinks, or excessive body muscle potentials occurred. The artifact rejection criterion was a peak–to–peak amplitude exceeding + /– 50 μV for EEG signal or ± 80 μV for EOG signal, and the rejection rate was ~ 6.0%. EEG sweeps related to incorrect behavioral responses (i.e., errors, FAs, and omissions) were also discarded from averaged ERPs, amounting, on average, to an average 8.1% (*SE* = 1.87) of rejected trials per participant. The minimum number of trials considered valid for analysis was 48. Before selective averaging, EEG signals were digitally filtered with a half–amplitude band-pass of 0.016–70 Hz. A time span of 500 ms divided cue presentation or omission from target administration, to which an EEG time span was added going from − 100 ms to 0 ms before cueing mode, which served as a baseline. The recording of a further 1000 ms time span followed target delivery, so to have a total of a 1600 ms EEG sweep length. As our focus was on investigating brain activation of attention processes, both phasic and tonic, in response to cue presentation or omission in various cue-target conditions (Fig. 1b and d), alongside examining the impact of hypoxia on pre-target preparatory states, also known as 'priming' states. Since the cue did not provide any information regarding the congruency-incongruency levels of the subsequent target-flanker string, EEG trials related to this factor were combined for statistical analysis. However, this was not done for the statistical analysis conducted on RTs.

Additionally, unlike what done previously by Zani et al.^15^, to investigate for potential differences in the attention shifting mechanisms across the horizontal meridian (HM) of visuospatial field in relation to the different cueing modes/tasks, EEG trials were also averaged as a function of target-related appearance location above-HM or below-HM. This way, separate cue-target average ERP waveforms were obtained not only for the two validly informative spatial cueing modes (i. e., LC and LCmot) either towards the AHM or the BHM visual field, but also for the alerting, but spatially uninformative cueing mode (i. e, CC) and neither alerting nor spatially informative cueing mode (i. e., NC) (Fig. 1b).

Again, to compare cue-related brain ERPs recorded in the double-choice motor-response condition (LCmot) with those generated by the cue in the cue-target single-choice motor-response conditions (i. e., CC, LC, and NC), ERPs to the different types of arrows (i.e., standard and star arrows) were also collapsed together in all the cueing conditions.

Hence, for each subject distinct ERP averages were obtained according to respiratory condition (i.e., ambient-air and hypoxia), cueing/task condition (CC, LC, NC, LCmot), location of the following visual target presentation in the visual field (above or below horizontal meridian (HM) of visuospatial field) following the cueing mode. Besides average ERP waveforms for each single participant, grand-average ERPs were also computed for the participants’ sample.

Furthermore, topographical voltage maps of ERPs were obtained by plotting color-coded isopotentials derived by interpolating voltage values between scalp electrodes as a function of respiratory and cueing conditions as well as target-related visual field location.

### Data analysis

#### Behavioral data

The behavioral literature shows how one approach to the study of alerting and attentional orienting is to use a warning signal (or cue) prior to a target event (e.g., Refs.^41,47^). If a speeded response to the target is required, reaction time improves following a warning. The literature also showed that the improvement in reaction time is accompanied by vast changes in the physiological state of the organism^22,23^. To further investigate differences between attentional alerting and orienting as well as the effects of hypoxia on these mechanisms, a four-way repeated-measures ANOVA was carried out on average reaction times (RTs). Factors used were respiratory condition (R, with two levels: Air and Hypoxia), cueing condition/task (T, with four levels: CC, LC, NC and LCmot), target spatial location (P, with two levels: Above and Below the visual field horizontal meridian (HM) and target vs flanker arrow-flankers congruency (C, Congruent and, I, Incongruent). Greenhouse–Geisser (ε) correction was carried out to compensate for possible violations of the sphericity assumption associated with factors which had more than two levels. The epsilon (ε) values and the corrected probability levels (in case of epsilon < 1) are reported. Post-hoc comparisons among means for significant factors with more than two levels were performed by means of Tukey HSD. The "Statistica" 10.0 package (Copyright^©^ by StatSoft Ltd, London, Beds MK 40 3EU UK, England) was used for the statistical analyses.

#### Electrophysiological data

**Figure 2:**
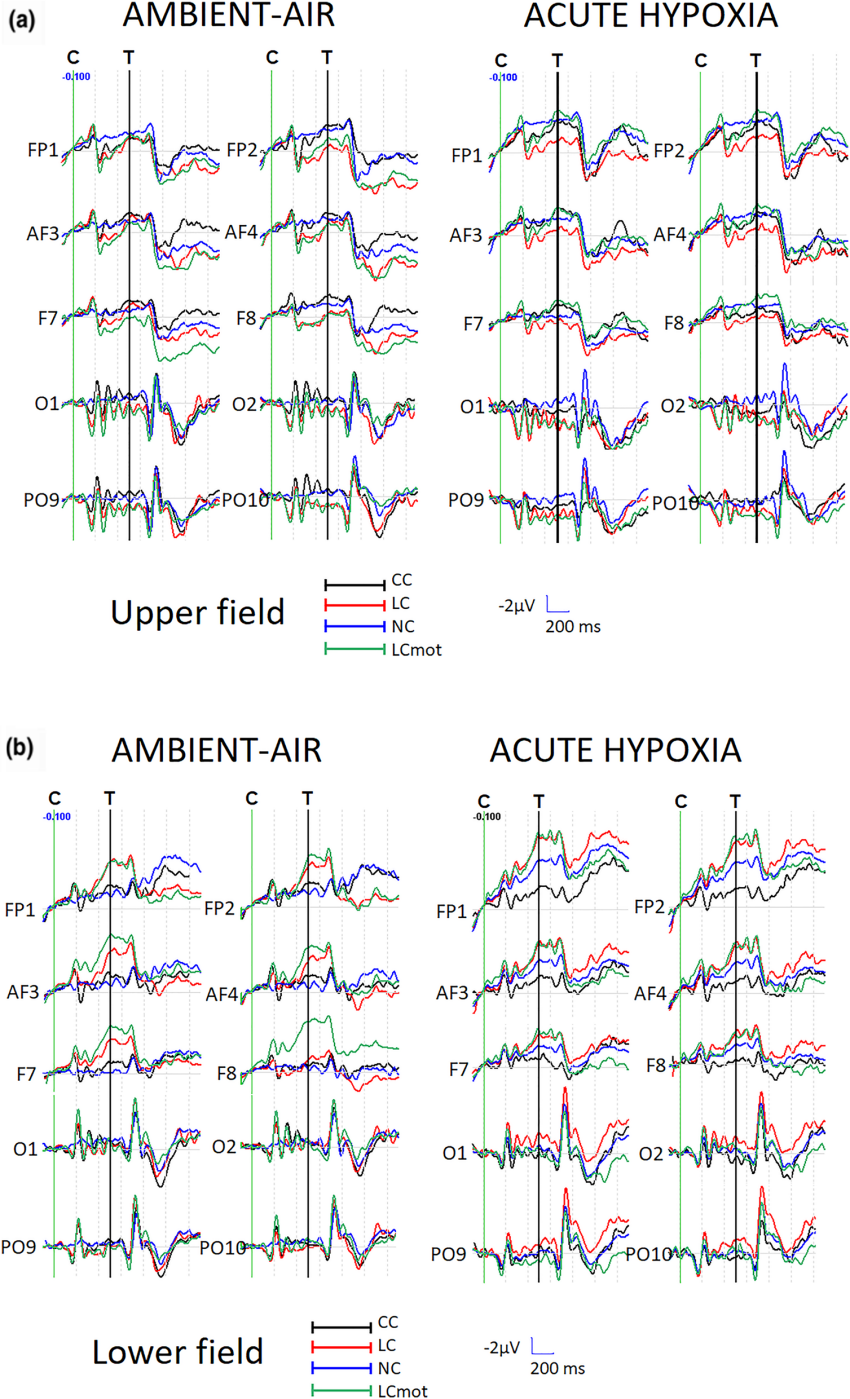
ERP waveforms grand averaged over the sample of subjects as obtained at left and right prefronto-polar (Fp_1_ and Fp_2_), mesial prefrontal (AF_3_ and AF_4_), dorsolateral prefrontal (F_7_ and F_8_), mesial occipital (O_1_ and O_2_), and parietal-occipital (PO_9_ and P_10_) electrode sites as a function of the two breathing conditions (ambient air and hypoxia), cueing/task mode (CC, LC, NC, LCmot), and target-related visual field (above-HM (**A**) and below-HM (**B**) target-related visual fields). Notably, a prominent, long-lasting negativity, consistent with an ADAN/CNV-like deflection, was recorded at anterior electrode sites between cue (C) and target (T) presentation, starting at ~ 220–250 ms post-cue. This negativity appears to be of greater amplitude in acute hypoxia than in ambient air, and in below-HM than in above-HM visual field conditions. Conversely, a long-lasting or tonic positivity (TP) can be observed at the posterior electrode sites, which is evident in the same time frame as the ADAN/CNV deflection and somewhat persists well after target presentation. This positivity, which is more pronounced for the above-HM than for the below-HM target-related condition, and of greater amplitude in hypoxia than in ambient air, is consistent with a LDAP/TP. Its changes in amplitude as a function of attentional cueing/task mode, among other factors, strongly support this view.

Figure 2A and B show the grand-average ERPs obtained at some representative anterior and posterior homologous scalp sites as a function of visual target-related presentation location in the visual field (i.e., above or below the visual field horizontal meridian (HM), respectively), following the prior valid cueing mode, and of respiratory condition (ambient-air vs. hypoxia). Visual inspection of the ERP waves recorded at the anterior electrodes shows an increasing ADAN/CNV slow negative potential compound, like the contingent negative variation, which developed between cue and target presentation and reached its greatest amplitude at prefronto-polar electrode sites (i.e., Fp_1_ and Fp_2_) in hypoxia for the below-HM target-related visual hemifield condition. Again, this negative drift appears of a different amplitude as a function of the cueing modes/tasks at the various mesial prefrontal and dorsolateral frontal electrode locations. Quite peculiarly, both the phasic alerting, but spatially uninformative, CC cueing mode and the utterly lacking any cueing before the wholly exogenous target-related attention-orienting NC mode showed a higher amplitude of the ADAN/CNV compound than both the phasic alerting and spatially informative LC and LCmot cueing modes in ambient-air. However, in hypoxia this finding is only true for LC but not for LCmot at the prefrontal and frontal scalp sites, where the above-HM target-related attention orienting is concerned (Fig. 2A again).

The mean amplitude of this ADAN/CNV compound was quantified in between 400 and 470 ms post-cue latency, but prior target administration, where it reached its maximum amplitude. Noteworthy, in the same post-cue, but prior-target latency range as for this negative slow wave, a late-latency long-lasting (or tonic) positivity (TP) quite like a LDAP can be appreciated in Fig. 2A and B over posterior scalp regions—e.g., the mesial-occipital (O_1_ and O_2_) and lateral occipito-parietal (PO_9_ and PO_10_) locations—mostly for the above-HM target-related visual field. Overall, our findings of negative and positive polarity slow potentials segregated at anterior and posterior scalp regions, respectively, fits well with the view of having recorded ADAN/CNV and LDAP/TP components, respectively, at these segregated scalp regions. Our findings fit also well with Kida’s and Imai’s^19^ original findings of a CNV-like negative slow wave (with frontal maximum) and a positive slow wave (with parietal maximum) following the ERPs P300, which progressively increased in amplitude as acutely simulated altitude increased from 0 m to 6000 m. However, unlike these original and at present almost neglected findings, the posteriorly centered late-latency LDAP/TP of greater amplitude in hypoxia than in ambient-air for the above-HM target-related visual hemifield found by us appears to change in amplitude as a function of cueing mode/task, as done by the ADAN/CNV negativity compound. Still more intriguingly, the LDAP/TP appears to show a reversed trend with respect to that shown by the ADAN/CNV compound. Indeed, the LDAP/TP for both phasic-alerting and spatially informative LC and LCmot cueing modes resulted in much larger amplitude than that for both fully spatially uninformative CC and NC modes. It is also most interesting that this positivity also seems to show much smaller amplitude in response to the different cueing modes/tasks and respiratory conditions for the below-HM target-related visual hemifield (Fig. 2B).

**Figure 3:**
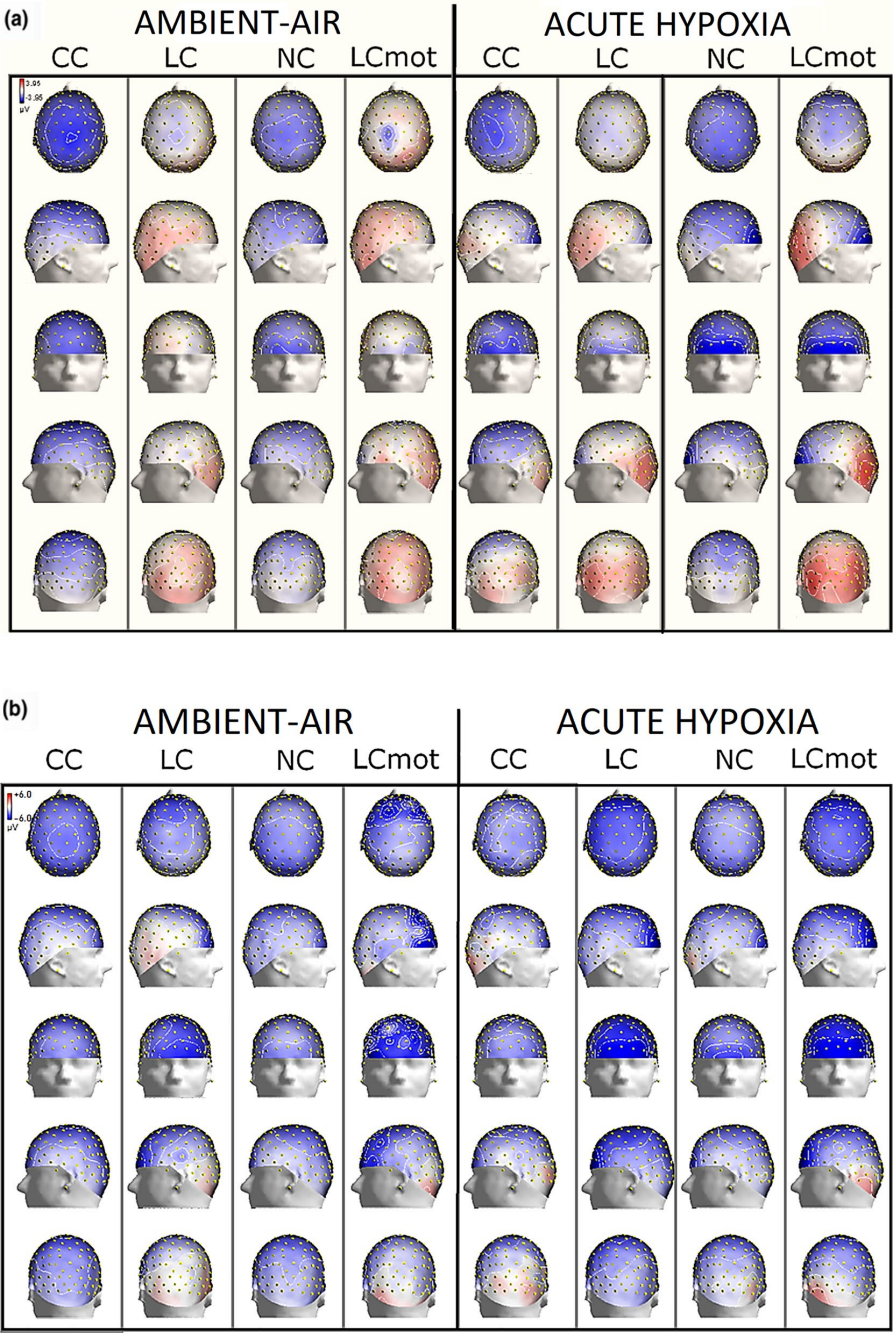
(**A**) 3D scalp maps of color-coded mean amplitude values computed in between 400–470 ms post-cue latency range with over imposed their relative contour-lines. The maps are plotted as a function of the CC, LC, NC and LCmot cuing/task modes in both the air and the hypoxia respiratory conditions for the “above-HM” target-related location. Note that, independent of the respiratory condition, for each cueing mode (reported along the columns in the figure) the maps illustrate the topographic distributions of scalp surface ERP voltages from the top, the right, the front, the left and the back views (as reported along the map rows), respectively. (**B**) Same as for (**A**) with the difference that the maps illustrate data obtained for the “below-HM” target-related location. Worth of note is that the scale size of isopotentials plotted for the “above-HM” target-related location is much smaller than that obtained for the “below-HM” target-related location, namely ± 3.95 µV Vs. ± 6 µV, respectively. As can be appreciated by the maps, this hints at overall larger ADAN/CNV mean values, but smaller LDAP/TP values, for the latter than the former target-related delivery location.

To investigate the possibly segregated topographic distribution of the anterior ADAN/CNV negative compound and of posterior LDAP/TP and possible differences across attentional cueing mode/tasks, target-related locations (upper vs. lower) and respiratory conditions, we computed isocontour voltage maps according to these factors by means of the spherical spline interpolation algorithm developed by Perrin et al.^53^. The measured voltages were then radially projected on the three-dimensional (3D) surface of an idealized head as drawn from the different top, right, front, left and back points of view as a function of the quoted factors (see Fig. 3A and 3B).

For control of statistical robustness of the differences noted in the spline maps as a function of respiratory condition, cueing mode/task and target-related cue location with respect to the visual field horizontal meridian (HM) as related to brain higher-order anterior and posterior neural connectivity and modulatory trends, cue-related ADAN/CNV compound mean amplitude values were measured in the 400–470 ms time window at representative, homologous mesial and dorsolateral prefronto-polar, prefrontal, frontal, and fronto-central electrode sites, namely, Fp_1_ and Fp_2,_ AF_3_ and AF_4,_ F_3_ and F_4_, F_7_ and F_8_, and FC_3_ and FC_4_. Conversely, the LDAP/TP mean area values were obtained in the same time window at homologous electrode sites over mesial and dorsolateral visual parietal-occipital, occipital and Inion scalp areas, namely, PO_3_ and PO_4_, O_1_ and O_2_, IN_1_ and IN_2,_ PO_7_ and PO_8_, and PO_9_ and PO_10_.

Mean amplitude measures thus obtained for these slow potentials were submitted to statistical analysis by means of two separate five-way repeated–measures ANOVAs with respiratory condition (R, 2 levels: Ambient-air or hypoxia), cueing condition/task (T, 4 levels: CC, LC, NC, and LCmot), location of visual target presentation in the visual field (L, two-levels: above or below HM of the visual field), hemisphere (H, two levels: left vs. right) and electrode (E, 5 levels: Electrode sites depending on the indicated ERPs component of interest) as factors. For significant effects of main factors with more than two levels and their interactions, post-hoc multiple comparisons were carried out by means of Tukey HSD. As for behavioral data, degrees of freedom were corrected, and p values adjusted when ε values were < 1. As for behavioral data, the "Statistica" 10.0 package was used for the statistical analyses of the electrophysiological measures.

### Institutional review board statement

The study was conducted according to the guidelines of the Declaration of Helsinki and approved by the Ethics Committee of National Research Council (CNR).

### Informed consent statement

Informed and written consent was obtained from all participants involved in the study.

### Study limits

One limit of the present investigation might be the relatively small sample size, so that further investigation might be needed to corroborate the present findings, which appear nevertheless solid from the methodologic point of view, besides from the robustness of its findings.

## Results

### Behavioural results

Importantly, the 4-way ANOVA yielded a significant finding [F(1,7) = 5.179; p < 0.0569; ε = 1] for the respiratory main factor, in that, in ambient-air participants gave an overall faster motor response (M = 478.25; SE = 14.470) than in hypoxia (M = 497.28; SE = 13.283). The ANOVA also indicated a significant target location factor [F(1, 7) = 6.695, p < 0.0334] per se, in that participants were overall quicker to respond to above-HM (M = 484 ms; SE = 12.30) than below-HM target-related visual hemifield (M = 492 ms; SE = 14.92), independent of all other factors. Interestingly, both target congruency and cueing/task main factors significantly affected subjects’ motor response as shown by a [F(1,7) = 163,053; p < 0,000004] for the former factor and by a [F(1,969; 13,779) = 74,415; ε = 0.661; adjusted p value < 0.0001] for the second factor, respectively. Indeed, a mean response speed of (M = 474 ms; SE = 12.904) was measured for congruent targets as compared to a longer time (M = 501 ms; SE = 13.509) for incongruent targets. As for the cueing mode/task main factor, pairwise comparisons indicated that, as compared to the other cueing modes, LCmot induced the slowest motor response (LCmot: M = 608 ms; SE = 18.716; CC: M = 454.28; SE = 14.84; LC: M = 418.60; SE = 13.42; NC: M = 469.39; SE = 15.04; p < 0.000000171 for all three pairwise contrasts) while, in turn, LC elicited the fastest response as compared with all the other cueing modes (p < 0.000001 for all three contrasts). Notwithstanding a consistent trend, CC did not induce a significant faster motor reaction time than NC, thus overall suggesting the neat efficient activation of the orienting network (CC-LC), but not of the alerting one (NC-CC).

Rather interestingly, the ANOVA also pointed out some two-way significant interactions. In fact, cueing mode/task speed was affected by respiratory condition [F(2,627; 18,339) = 4.449, ε = 0,876; adjusted p < 0.0185;]. Post-hoc contrasts carried out by Tukey tests for this interaction proved that, unlike CC, and NC cueing modes, the spatially informative cueing conditions (LC and LCmot) resulted in overall slower RTs in hypoxia than in ambient air (See Table 1, for mean RTs and S.E. as well as for p values for each pairwise comparison for these behavioral findings). Most importantly, further post-hoc contrasts across cueing modes, conducted separately for the two respiratory conditions, also revealed that, in air, LC resulted in faster reaction times than all the other cueing modes. Conversely, notwithstanding a tendency (p < 0.097), in hypoxia this speed advantage due to spatial-attention-orienting appears lost with respect to both CC and NC, but not LCmot. In turn, despite a tendency (i.e., p < 0.098) in ambient-air, apparently CC did not significantly speed up the reaction time as compared to NC neither in air nor in hypoxia, thus specifically hinting at a lack of consistent effects of respiratory regimen on alerting (See Table 1 again). The ANOVA also yielded a further significant two-way interaction of cueing mode/task x target-related location [F(1,427; 9.991) = 3,203; ε = 0.69, adjusted p < 0,0441], regardless of respiratory condition and target congruency. The pairwise contrasts for this interaction demonstrated that, unlike for CC mode, RTs to above-HM targets were faster than those to below-HM targets for all the other three cueing modes. Furthermore, positive effects of alerting could be seen for the below-HM target-related location, but not for the above-HM target-related one (see Table 2 for mean RTs and S.E. as well as for p values for each pairwise comparison).

**Table 1:** Mean reaction times (RTs) and relative standard errors (S.E.) computed over the subjects’ sample as a function of respiratory mode (Resp. Mode) and cueing mode/task (C/T Mode), independent of target-related location and target congruency.

Resp. mode	C/T mode			
	CC	LC	NC	LCmot
Ambient-air	443.45	399.63	469.92	591.07
	13.07	14.17	14.45	22.12
Hypoxia	465.10	437.57	478.85	626.52
	20.38	14.18	17.53	18.20

Results of post-hoc statistical paired contrasts with Tukey tests for ANOVA				
Ambient-Air	Hypoxia	Air vs. Hyp		
CC vs. LC = p < 0.0134	CC vs. LC = n. s	CC_Air_ vs. CC_Hyp_ = n. s		
CC vs. NC = n. s	CC vs. NC = n. s	LC_Air_ vs. LC_Hyp_ = p < 0.0059		
CC vs. LCmot = p < 0.0001	CC vs. LCmot = p < 0.0001	NC_Air_ vs. NC_Hyp_ = n. s		
LC vs. NC = p < 0.0001	LC vs. NC = n. s	LCmot_Air_ vs. LCmot_Hyp_ = p < 0.009		
LC vs. LCmot = p < 0.0001	LC vs. LCmot = p < 0.0001			
NC vs. LCmot = p < 0.0001	NC vs. LCmot = p < 0.0001

**Table 2:** Mean reaction times (RTs) and relative standard errors (S.E.) calculated on the sample of subjects as a function of cueing/task mode (C/T Mode) and above-HM (AHM) or below-HM (BHM; T. location) target location in the visual field, regardless of respiratory regime and target congruence.

T. location	C/T Mode			
	CC	LC	NC	LCmot
Above-HM (AHM)	454.45	413.81	462.06	604.03
	13.47	12.74	14.12	18.03
Below-HM (BHM)	454.10	423.40	476.71	613.57
	16.75	14.25	16.18	19.43

Results of post-hoc statistical pairwise contrasts with Tukey tests for ANOVA				
AHM vs. BHM	Below-HM (BHM)	Above-HM (AHM)		
CC_AHM_ vs. CC_BHM_ = n. s	CC vs. LC = p < 0.00001	CC vs. LC = p < 0.00001		
LC_AHM_ vs. LC_BHM_ = p < 0.048101	CC vs. NC = p < 0.00002	CC vs. NC = n. s		
NC_ABM_ vs. NC_BHM_ = p < 0.00620	CC vs. LCmot = p < 0.00001	CC vs. LCmot = p < 0.00001		
LCmot_AHM_ vs. LCmot_BHM_ = p < 0.01051	LC vs. NC = p < 0.00001	LC vs. NC = p < 0.00001		
	LC vs. LCmot = p < 0.00008	LC vs. LCmot = p < 0.00001		
	NC vs. LCmot = p < 0.00001			

Among significant behavioral effects highlighted by the ANOVA there was also the two-way interaction of cueing/task x target congruency [F(2,036; 14,527) = 19.374, ε = 0.679; adjusted p < 0.00001]. Post-hoc comparisons showed that reaction times were overall faster to congruent than incongruent targets for the whole of CC, LC, and NC modes, but not for LCmot cueing mode, very probably for an overall motor choice time ceiling effect. Moreover, further pairwise contrasts indicated that, no matter target congruency, LC induced the fastest RTs as compared to the other cueing modes/tasks, while, in turn, CC elicited faster RTs than both NC and LCmot, and NC faster RTs than LCmot (see Table 3 for a summary of these findings).

**Table 3:** Mean reaction times (RTs) and relative standard errors (S.E.) computed over the subjects’ sample as a function of cueing/task mode (C/T Mode) and target congruency (Cong = Congruent; Incong = Incongruent), regardless of target-related location and of respiratory condition factors.

Target congruency	C/T Mode			
	CC	LC	NC	LCmot
Congruent	437.51	437.51	449.95	605.04
	14.32	12.98	13.96	18.63
Incongruent	471.04	430.78	488.82	612.56
	15.70	13.98	16.22	18.86

Results of post-hoc statistical paired contrasts with Tukey tests for ANOVA				
Congruent target	Incongruent target	Cong vs. Incong		
CC vs. LC = p < 0.00015	CC vs. LC = p < 0.00015	CC_Cong_ vs. CC_Incong_ = p < 0.00015		
CC vs. NC = p < 0.01158	CC vs. NC = p < 0.00035	LC_Cong_ vs. LC_Incong_ = p < 0.00015		
CC vs. LCmot = p < 0.00015	CC vs. LCmot = p < 0.00015	NC_Cong_ vs. NC_Incong_ = p < 0.00015		
LC vs. NC = p < 0.00015	LC vs. NC = p < 0.00015	LCmot_Cong_ vs. LCmot_Incong_ = n. s		
LC vs. LCmot = p < 0.00015	LC vs. LCmot = p < 0.00015			
NC vs. LCmot = p < 0.00015	NC vs. LCmot = p < 0.00015			

### Electrophysiological results

#### Topographical mapping

As clearly visible in Fig. 3A and B, overall spline maps confirmed that, no matter the respiratory condition considered, with some differences across cueing modes/tasks, the topographic distribution of the ADAN/CNV was mostly delimited to the anterior prefronto-polar, prefrontal, and frontal scalp areas as well as to superior parietal scalp areas. Importantly, this negative slow potential also appears to change in response to respiratory condition and to attention cueing/task modes, showing, overall, a rather compound pattern. Indeed, the maps show that this ADAN/CNV compound greatly increased in amplitude for the below-HM visual hemifield with respect to the above-HM hemifield. Furthermore, it appears to reach much higher amplitudes for the spatially uninformative cueing/task modes (i.e., CC and NC) than for the spatially informative ones (i.e., LC and LCmot), when the above-HM target-related visual hemifield is considered. Conversely, it presents a higher amplitude for the spatially informative cueing/task modes (i.e., LC and LCmot) than for the spatially uninformative ones (e.g., CC and NC), where the below-HM target-related visual hemifield is concerned.

Regarding the LDAP/TP, the topographical maps indicate that the latter was more dominant in the posterior parietal, occipital, and temporal scalp regions. This trend was consistently observed regardless of respiratory conditions, particularly for the above-HM target-related visual field and for both spatially informative attentional cueing modes/tasks, that is LC and LCmot. Overall, this suggested that these positive slow potentials, which arose in between cue and target stimuli, may mirror a reflexive cue-triggered, pre-target modulation of visual brain areas activation (or an attentional priming) for boosting the processing of following target stimuli at the validly cued spatial location. Interestingly, biased activation is more pronounced in hypoxia than in ambient air, particularly for the location related to the above-HM target. Despite showing impressively much lower amplitude, this trend could also be seen for the below-HM target-related location (Fig. 3A).

##### ADAN/CNV compound (400–470 ms)

**Figure 4:**
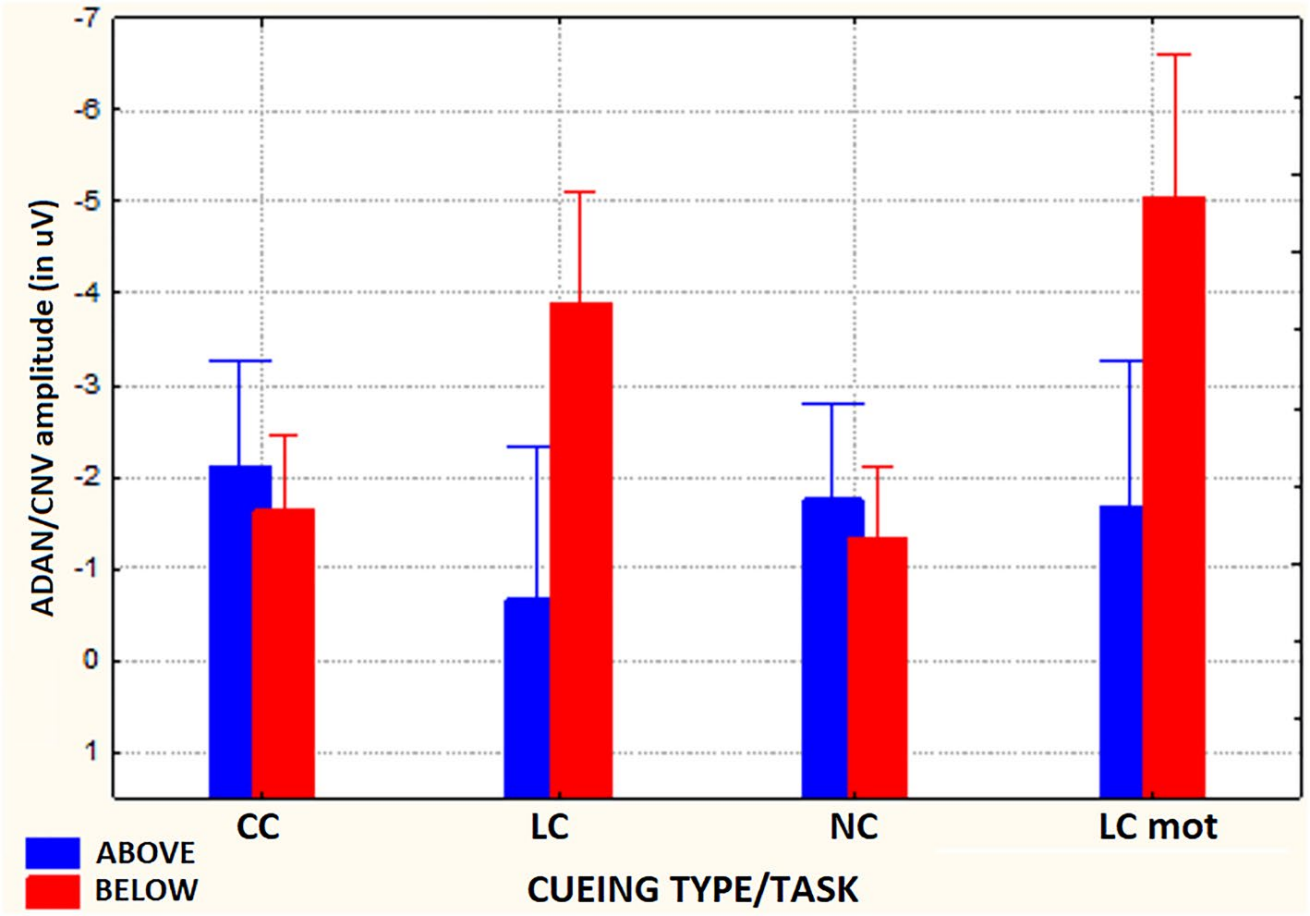
Amplitude of ADAN/CNV variation as recorded for the above Vs. below visual target-related locations as a function of the cueing/task modes, independent of respiratory conditions and electrode sites. Note that the scale of values is drawn with the same polarity trend as for the drawn waveforms, that is, with negativity up and positivity below. Note also that this applies for both the components analyzed and for all the figures reported in the paper.

The ANOVA carried out on mean area amplitude of ADAN/CNV-like deflection yielded the significance of the main cueing/task factor [F(2,59; 18,16) = 9.23, ε = 0.865, adjusted p < 0.0009]. Relative post-hoc comparisons showed that this negative slow potential was greatest (p < 0.005) for LCmot cueing condition (M = − 3.36 µV, SE = 0.56) with respect to the other conditions (CC: M = − 1.88 μV, SE = 0.38 (p < 0.0004); LC: M = − 2.28 μV, SE = 0.5 (p < 0.0328); NC: M = − 1.53, SE = 0.31 μV, p < 0.0001). In turn, LC proved to induce a larger ADAN/CNV than NC (p < 0.0014), but not than CC (n. s.), while, again, the slow negativity elicited by CC was not higher than that elicited by NC (n. s.). Overall, these findings suggested that, regardless of other variables, attentional-orienting network was only active at greater preparatory workload levels (LCmot vs. CC) and that, conversely, attentional-alerting network was not affected at all. Furthermore, the significance of target-related location factor [F(1,7) = 11.93, p < 0.01, ε = 1] confirmed that ADAN/CNV was overall much greater for below horizontal-meridian (HM) (M =  − 2.97 μV, SE = 0.41) than for above-HM (M =  − 1.55 μV, SE = 0.47) targets-presentation location. The ANOVA also yielded the significance of electrode factor [F(1,32; 9,32) = 31.5, ε = 0.33, adjusted p < 0.0001]. Post-hoc pairwise contrasts showed that the ADAN/CNV was greatest over prefronto-polar sites (FP_1/2_: M = -3.74 μV, SE = 0.54) with respect to all the other anterior sites (AF_3/4_: M =  − 2.37 μV, SE = 0.45; F_7/8_: M =  − 1.45 μV, SE = 0.38; F_3/4_: M =  − 1.92 μV, SE = 0.36; FC_3/4_: M =  − 1.83 μV, SE = 0.28), and that, in turn, was significantly greater at AF_3/4_ than at F_7/8_. The ANOVA also yielded the statistical significance of cueing/task x target-related location factors [F(2,1; 14,7) = 20.9, ε = 0.7, adjusted p < 0.00004]. Post-hoc comparisons demonstrated that, no matter respiratory condition and electrode, ADAN/CNV was greater for the below-HM than the above-HM condition for both the spatially informative LC (p < 0.0001) and LCmot (p < 0.001) cuing/task modes (see Fig. 4) for means and standard errors). According to Tukey post-hoc contrasts across cueing modes for the below-HM target-related location, ADAN/CNV was greatest to LCmot than for all the other cueing modes; in turn, it was significantly larger for LC than both CC and NC cueing/task modes. Conversely, overall, ADAN/CNV to CC mode did not differ from that to NC mode neither above-HM nor below-HM, thus hinting at a deficiency of attention alerting for both the target-related locations. Furthermore, for the above-HM target-related condition ADAN/CNV was only larger for CC than LC cueing condition (p < 0.05).

Also significant was the interaction of target-related location x hemisphere [F(1,7) = 14.44, p < 0.007, ε = 1] which showed no hemispheric asymmetry for the amplitude of ADAN/CNV as the above-HM condition (LH: M = – − 1.61 μV, SE = 0.43; RH: M =  − 1.51 μV, SE = 0.51 μV) was concerned and a clear left-sided hemispheric lateralization of it for the below condition (LH: M =  − 3.18 μV, SE = 0.41; RH: M =  − 2.76 μV, SE = 0.43).

**Figure 5:**
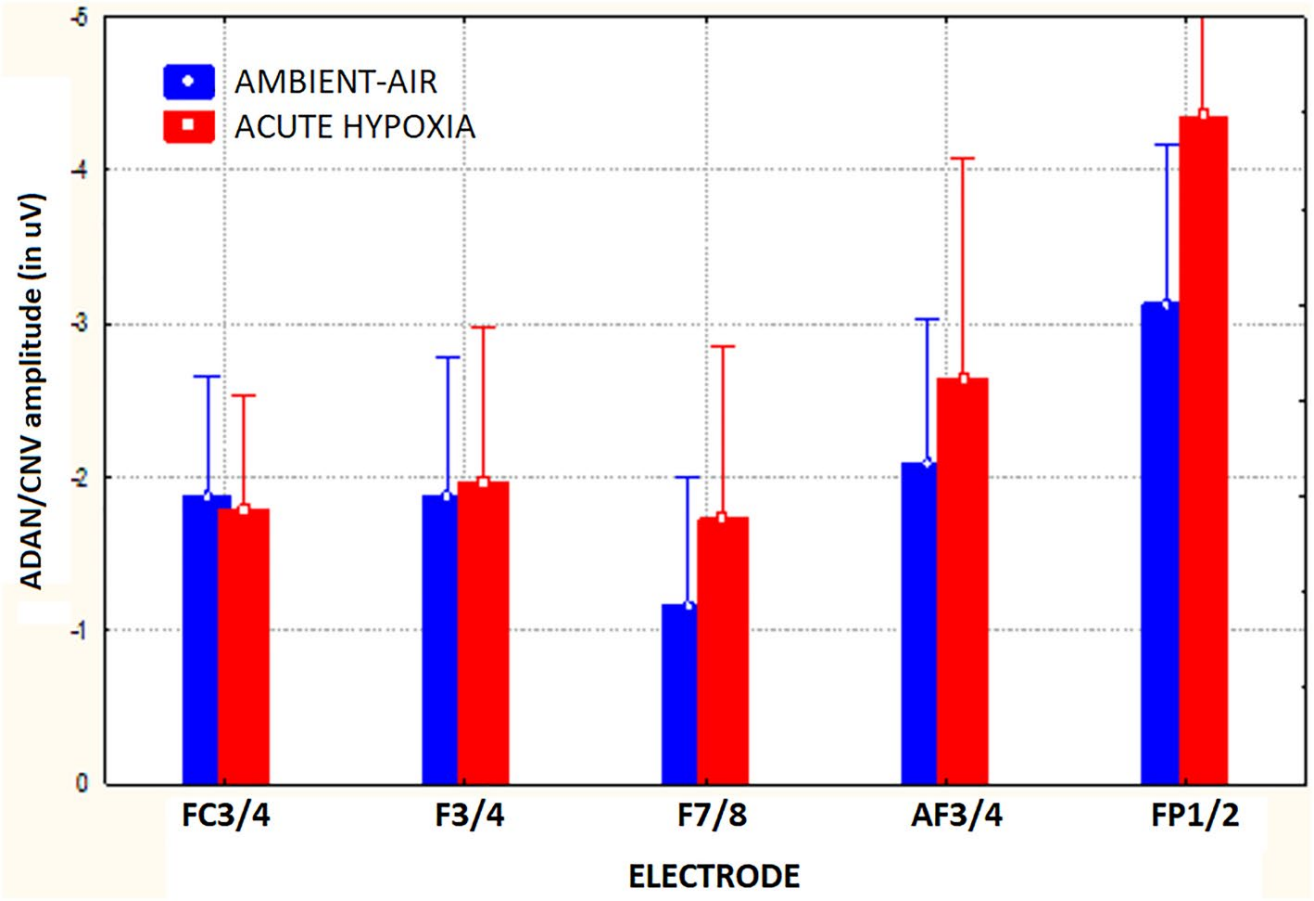
Mean amplitude values of ADAN/CNV recorded at anterior electrode sites as a function of respiratory conditions, independent of attentional cueing/task mode and target location.

Additionally, ADAN/CNV gave rise to the significant interaction of respiratory condition x electrode [F(2,6; 18,2) = 3.602; ε = 0.65, adjusted p < 0.0450]. Tukey Post-hoc comparisons showed how ADAN/CNV was larger in hypoxia (M =  − 2.50 μV, SE = 0.51) than in ambient-air (M =  − 2.03 μV, SE = 0.35) at dorsolateral, prefrontal, and fronto-polar scalp sites (F_7/8_, p < 0.04; AF_3/4_, p < 0.05; and FP_1/2_, p < 0.0002), as displayed in Fig. 5.

**Figure 6:**
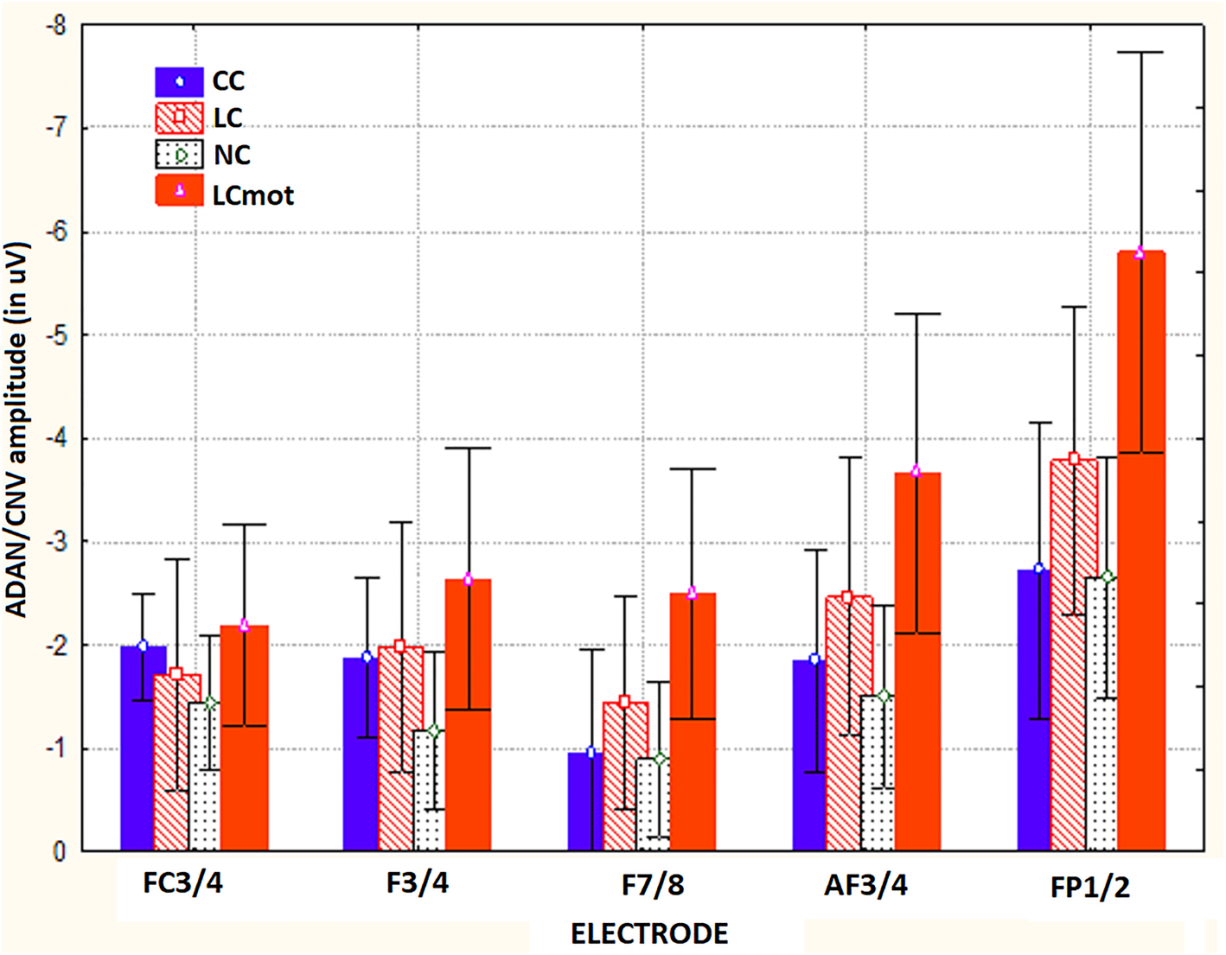
Mean amplitude values of ADAN/CNV deflection recorded at anterior scalp sites as a function of cueing/task conditions, independent of respiratory conditions and of target-related delivery location.

Also significant was the interaction of cueing/task mode x electrode [F(3,36; 23,51) = 8.71, ε = 0.28, adjusted p < 0.0003], independent of respiration mode and target-related position. Figure 6 shows ADAN/CNV mean amplitude values (along with standard errors) relative to this interaction. Tukey post-hoc comparisons showed that at both superior, medial-dorsolateral FC_3/4_ and F_3/4_ scalp sensors, ADAN/CNV compound did not show neither alerting nor orienting effects, although at the latter sensor, the response elicited by LCmot was larger than that for all the other cueing conditions (p < 0.001 for all the pairwise contrasts). Conversely, at more anterior, superior dorsomedial AF_3/4_ sites and inferior dorsolateral F_7/8_ sites as well as prefronto-polar Fp_1/2_ scalp sites LCmot elicited a higher ADAN/CNV than all the other LC, CC, and NC cueing conditions (p < 0.001 for all the pairwise contrasts); in turn, LC induced a larger ADAN/CNV than the CC and NC cueing modes/tasks (p < 0.0001 for both the pairwise contrasts). Notably, no alertness effect was found, not being the negativity for CC more effectual than that for NC at all the latter electrodes.

**Figure 7:**
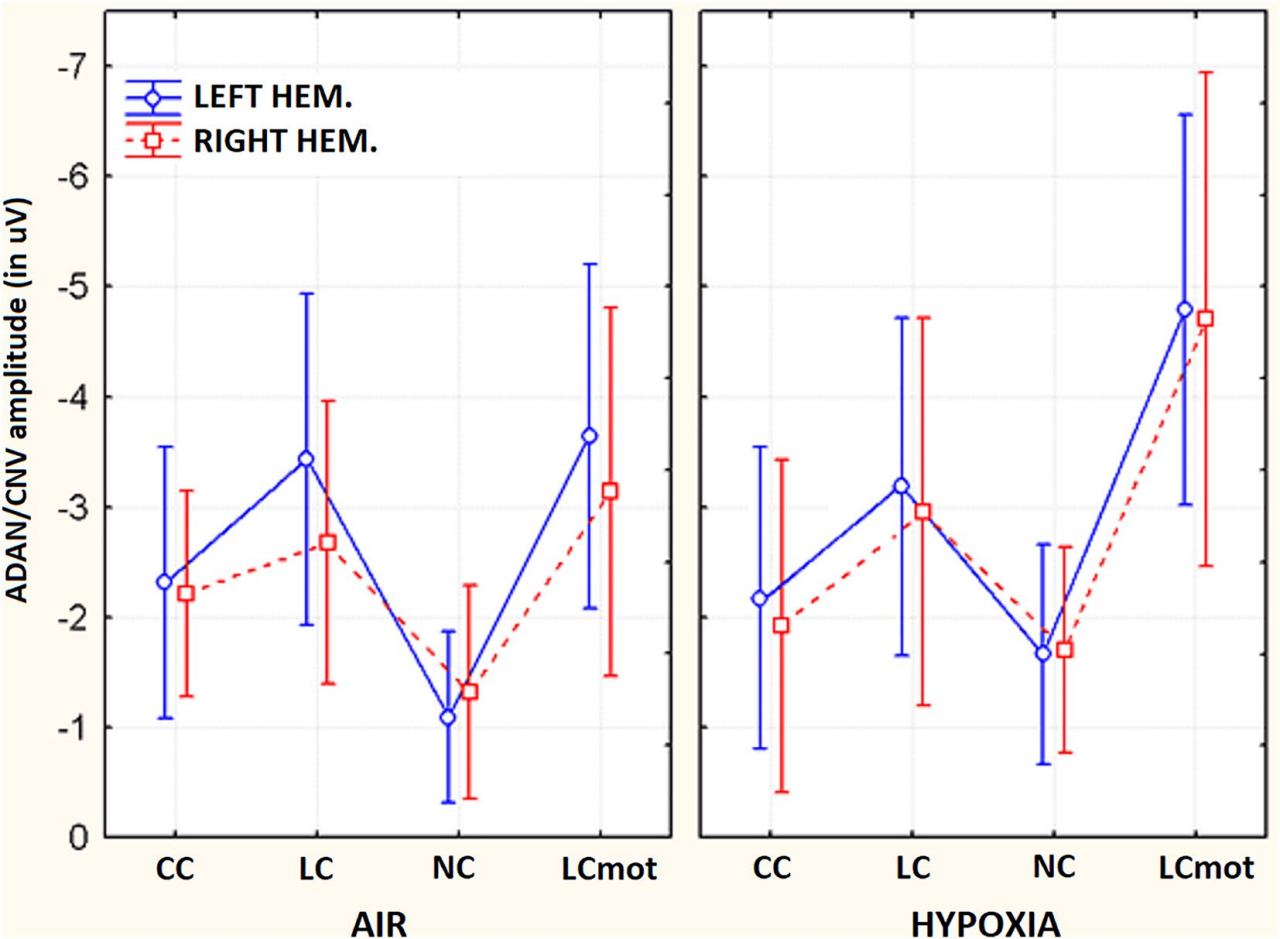
Mean amplitude values of ADAN/CNV response recorded as a function of respiratory condition, cueing mode/task and brain hemisphere.

As shown by post-hoc comparisons for the significant interactions of location x electrode [F(1,4; 9,8) = 10.86, ε = 0.35, adjusted p < 0.005] and of cueing/task mode x location x electrode [F(1,56; 10,92) = 7.45, ε = 0.13, adjusted p < 0.01], the influences of spatially informative cueing/task conditions on anterior negativity were especially appreciated for the below-HM target-related visual hemifield at prefronto-polar, medial prefrontal and dorsolateral prefrontal electrode sites. The post-hoc contrasts carried out for both the 3-way interaction of respiratory condition x cueing/task x hemisphere [F(3,21) = 3.812; p < 0.025; ε = 1] and the 2-way interaction of respiratory condition x hemisphere [F(1,7) = 5.91; p < 0.045; ε = 1] indicated a larger increase of ADAN/CNV amplitude over the right-sided anterior scalp regions in hypoxia than in ambient-air for both the LC and LCmot alerting and spatially-informative cueing/task conditions (Air: LH, M =  − 2.188 μV, SE = 0.33; RH: M =  − 1.87 μV, SE = 0.38; Hypoxia: LH, M =  − 2.59, SE = 0.47; RH, M =  − 2.4 μV, SE = 0.56) (see Fig.). As a result of the latter increase in hypoxia, the left-sided hemispheric asymmetry for ADAN/CNV amplitude found for these spatially orienting cueing modes in air could not be appreciated in hypoxia (Fig. 7).

As concerns the ADAN/CNV mean amplitude values, a further relevant finding was the 3-way interaction of respiratory mode with the cueing mode/task and the target-related presentation location factors, regardless of electrode and hemisphere factors (F(3,21) = 4.027; p < 0.020). Post-hoc analyses for this ANOVA result proved that, as compared to the amplitudes measured for CC and NC cueing conditions, which, no matter the respiratory regimen, did not differ from each other, the amplitudes of this late negativity for LC and LCmot showed to be noticeably larger in relation to the below-HM target-related delivery location, during the oxygen deficiency condition. Due to these trends, CC showed to be related to a greater ADAN/CNV than the other LC, NC and LCmot modes for above-HM target-related location in ambient-air. Intriguingly, in this same respiratory condition, CC showed a lower ADAN/CNV than LC and LCmot modes for the below-HM target-related presentation location.

**Figure 8:**
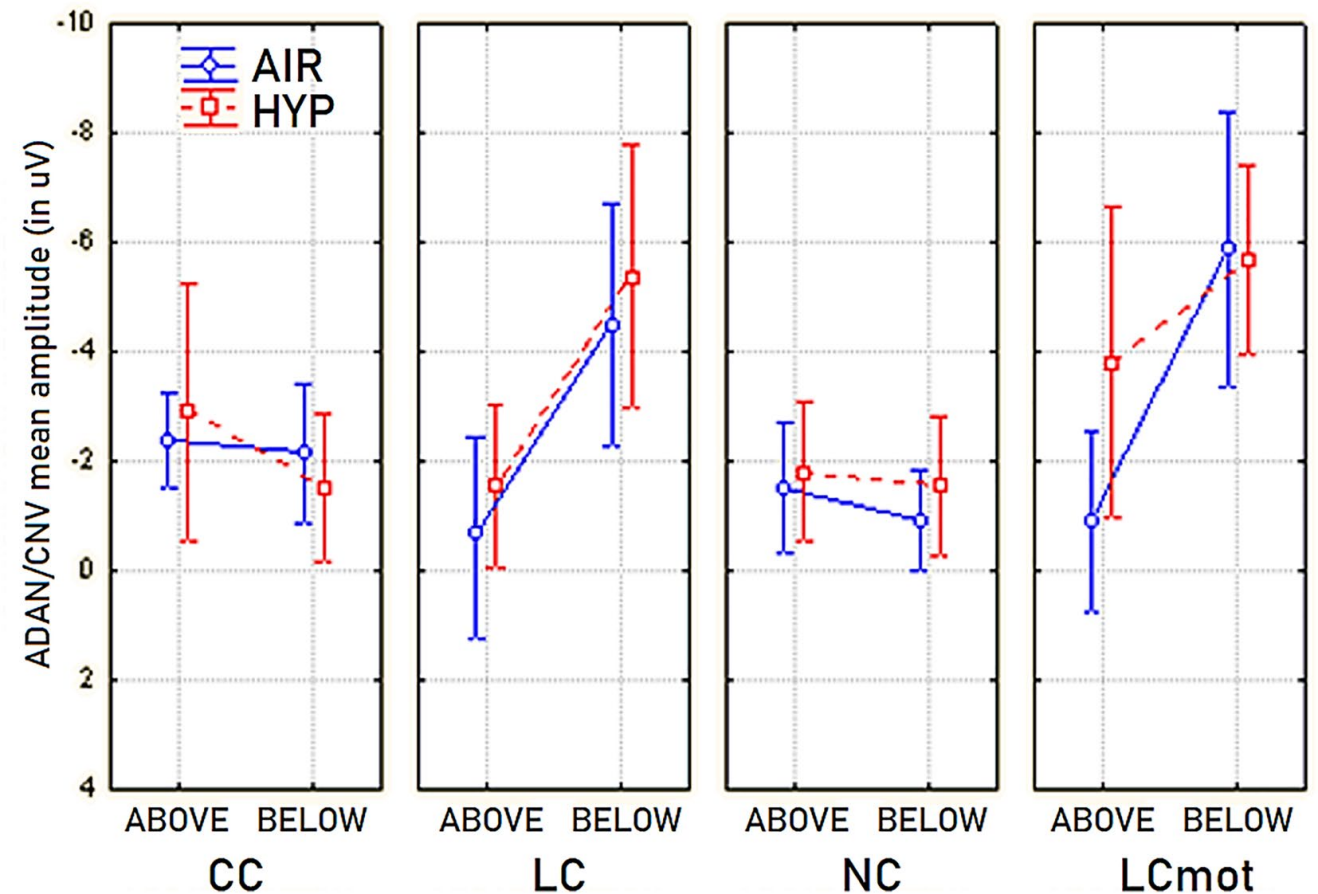
Mean amplitude values of ADAN/CNV response measured as a function of respiratory condition, cueing/task mode and target-related delivery location.

With a similarly complex trend, LC obtained a lower ADAN/CNV than LCmot, CC, and NC for above-HM target-related presentation location in hypoxia; in turn, LCmot resulted in a lower ADAN/CNV than both CC and NC for the above-HM target-related location during the acute hypoxic condition. Finally, during the shortage of oxygen session both LC and LCmot showed a much greater ADAN/CNV amplitude than both CC and NC for the below-HM target-related presentation location (See Fig. 8).

##### LDAP/TP (400–470 ms)

The ANOVA performed on the mean amplitude of the LDAP/TP yielded the significance of location [F(1,7) = 10.24; p < 0.01], with greater amplitudes of LDAP/TP for the above (M = 0.587 μV, SE = 0.256) than below-HM target-related condition (M = -0.501 μV, SE = 0.37). Also significant was the electrode factor [F(4,28) = 4.73; p < 0.005], with greater amplitudes at lateral occipito-parietal (PO_9_/PO_10:_ M = 0.39 μV, SE = 0,192) than mesial occipital (O_1_/O_2_: M = 0.166 μV, SE = 0.23) or inion (IN_1_/IN_2_: M = − 0.32 μV, SE = 0.36) sites.

Furthermore, ANOVA yielded the significance of the location x electrode interaction [F (4,28) = 3.95; p < 0.01]. Relative post-hoc comparisons indicated how LDAP/TP was larger to above than the below visual-HM condition at all electrode sites.

**Figure 9:**
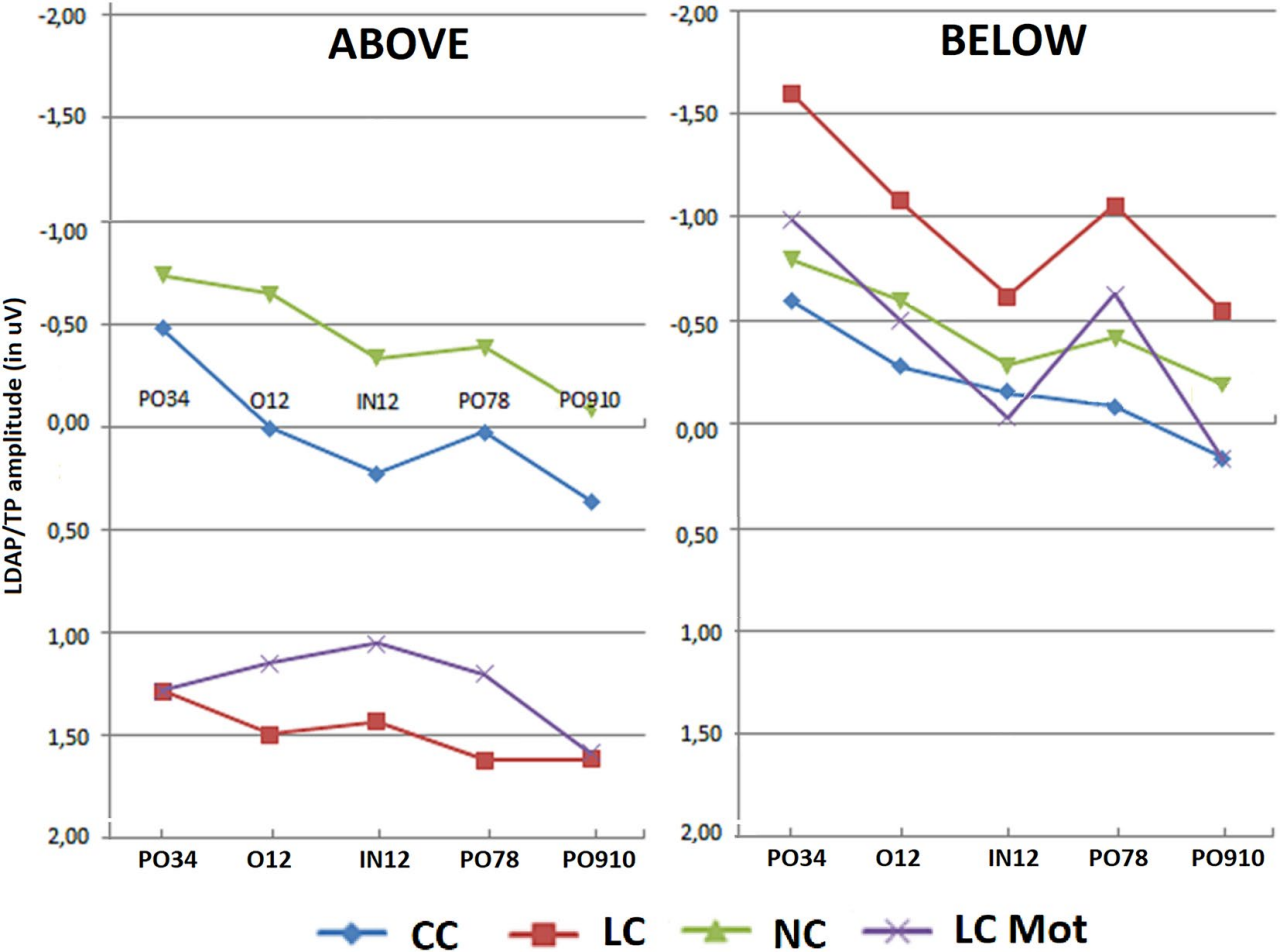
Mean amplitude of *LDAP/TP* recorded at posterior scalp sites as a function of cueing/task modes, target stimulus delivery location, and electrode sites, regardless of respiratory conditions.

Worth noting is also that the positivity recorded at the visual areas changed as a function of the co-occurring influences of cueing mode/task and target-related location [F(3,21) = 3.617; p < 0.022], with a significantly (p < 0.03) larger LDAP/TP to local spatially informative cueing (LC: M = 1.4954 μV, SE = 0.35) than no cueing (NC: M =  − 0.44 μV, SE = 0.4) for the above-HM condition only. This was apparently due to the differential effects of cueing location factor found at the different electrode sites. Indeed, the significant cueing/task x location x electrode interaction [F(12,84) = 3.67; p < 0.0001] and the relative single pairwise contrasts showed a significant modulation of LDAP/TP due to cueing/task conditions, with a larger positivity to LC than LCmot for the above-HM visual hemifield at Inion (IN_1/2_; p < 0.05) and lateral-occipital (PO_7/8,_ p < 0.05) sites. LC stimuli also elicited a much larger LDAP/TP (p < 0.001) than both CC and NC at all electrode sites (Fig. 9). As for the effect of alertness (deriving from the comparison between the “central cue” (CC) mode with the “no cue” one, NC), LDAP/TP revealed to be greater to CC than NC conditions at O_1/2_, IN_1/2_, and PO_9/10_ sites (p < 0.001 for all three electrode sites). Conversely, spatial orienting (LC) attentional cueing toward the below-HM target-related visual hemifield elicited a smaller LDAP/TP than both CC and LCmot, besides than NC (p < 0.001), at all electrode sites (p < 0.001 for all the pairwise comparisons), except for the ones over Inion (IN1/2) and more lateralized parietal-occipital scalp regions, namely, PO9/10. For the below-HM target-related location, pairwise contrasts also indicated that there were no significant alerting differences in the LDAP/TP amplitude elicited by CC vs. NC conditions.

The further interaction of electrode x hemisphere [F(4,28) = 3.78; p < 0.014] indicated how LDAP/TP was asymmetrically distributed over the right hemisphere, especially at PO_3−4_ sites (PO_3_: M =  − 0.624, SE = 0.41; PO_4_: M =  − 0.02 μV, SE = 0.33).

The post-hoc contrasts for the significant cueing/task x location x hemisphere three-way interaction [F(1,7) = 7.04; p < 0.03] indicated that the LDAP/TP was greater over the right than the left hemisphere only for the CC condition as the below-HM target-related condition of the location factor was concerned (LH: M =  − 0.7609 μV; RH: M = 0.0719 μV; p < 0.003).

**Figure 10:**
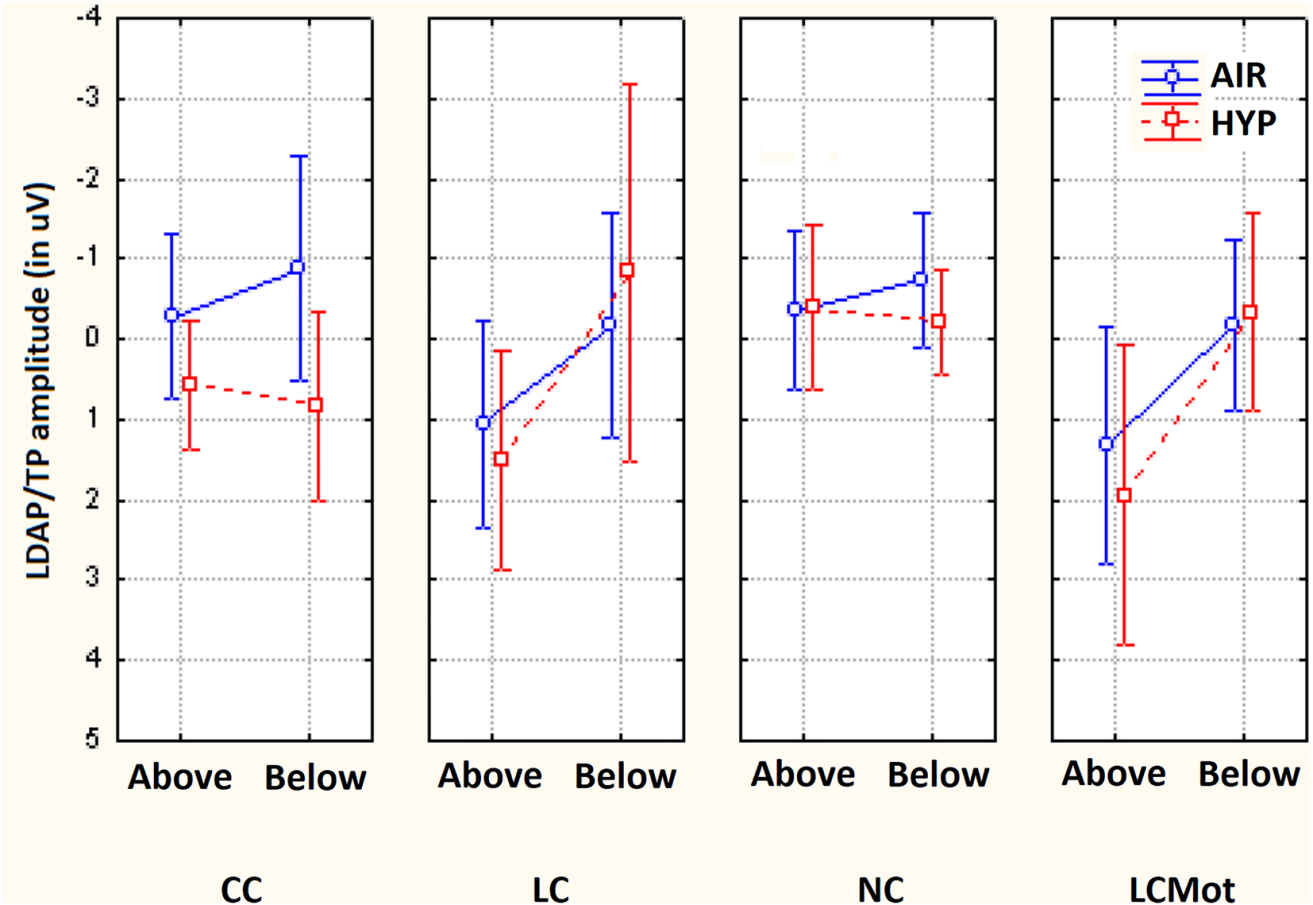
Mean amplitude of *LDAP/TP* obtained at posterior scalp regions as a function of respiratory condition, cueing mode/task, and target-related stimulus delivery locations, regardless of electrode site considered.

As for effects of brain oxygenation levels at visual occipital scalp sites, the ANOVA yielded a most interesting triple interaction of the respiratory condition x cueing mode/task x target-related location (F(3,21) = 4.18; p < 0.030), which mirrored the similar 3-way interaction for ADAN/CNV. According to post-hoc pairwise contrasts this triple interaction indicated that for above-HM target-related delivery location in ambient air, CC and NC hardly showed any LDAP/TP response whose amplitude did not show any difference from each other. Intriguingly, both LC and LCmot indicated to be related to higher LDAP/TP amplitudes than both CC and NC (p < 0.0001 for all the pairwise comparisons) for the default respiratory regimen and target-related location indicated above (see Fig. 10).

Conversely, for the below-HM target-related location, within pairwise contrasts for hypoxia condition indicated that CC elicited a higher PDAP/TP than LC, NC, and LCmot cueing modes (p < 0.00001 for all the pairwise contrasts). This late positivity also showed a knotty trend of amplitude changes across cueing mode/tasks as a function of target-related delivery location. Namely, LDAP/TP not only decreased dramatically in amplitude from the above-HM to the below-HM target-related location, but also across cueing mode/task (Fig. 10 again).

In addition, the ANOVA also indicated that the respiratory condition affected both the location and the hemisphere factors [F (1,7) = 6.99; p < 0.03]. Pairwise comparisons showed a strong effect of hypoxia, with overall smaller LDAP/TP during this respiratory condition than in ambient-air (hypoxia =  − 0.17 µV, SE = 0.34; air = 0.25 μV, SE = 0.29; p < 0.001) for the below-HM target-related location condition. For the above-HM location, post-hoc comparisons revealed that an acute oxygen respiratory deficiency specifically increased the LDAP/TP over the right (RH: Air = 0.41 μV vs. Hypoxia = 1.26 μV; p < 0.0006) but not the left hemisphere (LH: Air =  − 0.37 vs. Hypoxia =  − 0.98; n. s.).

### Source reconstruction analyses

**Figure 11:**
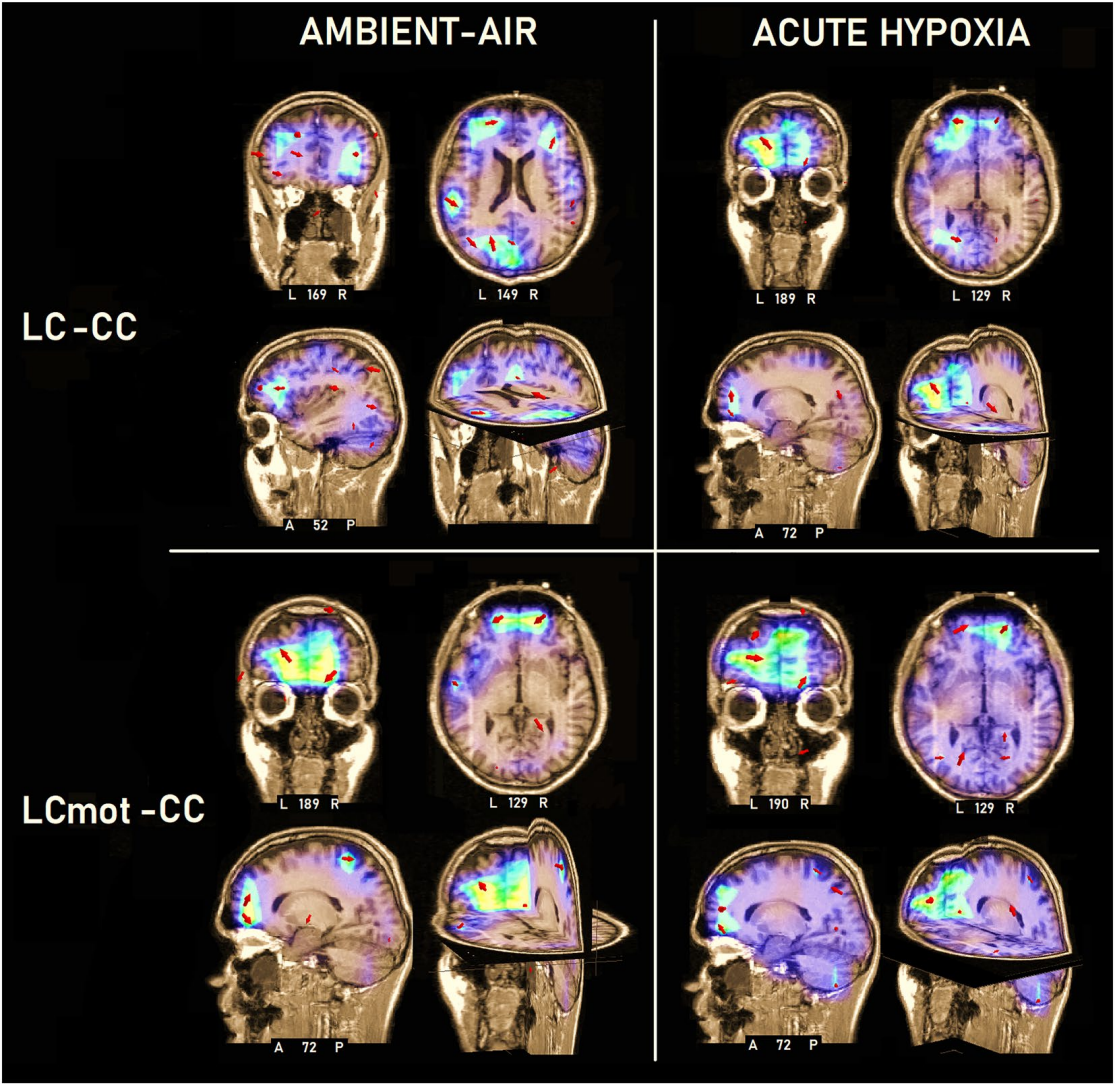
Inverse solutions computed with sLORETA for (A) the difference between LC and CC in ambient air (LC–CC air) and (B) between the same cueing conditions in acute hypoxia (LC–CC hyp). (C) the difference between LCmot and CC in ambient air (LCmot–CC air), and (D) that between the indicated cueing conditions in hypoxia (LCmot–CC hyp), in the 400–470 ms pre-target time range.

Since it was among our most relevant goals to investigate how brain areas subserving attentional shifting of the focus of spatial attention in space were affected by hypoxia, a sLORETA inverse solution (Fig. 11) was applied to the difference of ERP responses recorded in both the phasic alerting and orienting informative conditions (i. e., LC and LCmot) and the tonic alerting but spatially uninformative one (i.e., CC) in the post-cue latency range of 400–470 ms prior target presentation, separately for ambient-air and hypoxia. Table 4 reports the electromagnetic dipoles that most probably generated the surface voltage of both the ADAN/CNV and LDAP/TP components in both orienting conditions and respiratory conditions.

**Table 4:** List of active sources according to sLORETA explaining the difference voltage between the central uninformative condition (CC) and the two spatially informative cueing conditions [i. e., (LC–CC) and (LCmot–CC)], respectively, computed in between 400 and 470 ms post-cueing time window (i. e., ADAN/CNV and LDAP/TP).

Magn	T–x	T–y	T–z	Hem	Lobe	ROI	Gyrus/Area	BA
(LC–CC) attentional cueing in ambient-air								
12.126	− 20	− 60	44	L	P		Pre-Cun	7
12.126	− 20	− 60	44	L	P	2 mm	SPL	7
12.126	− 20	− 60	44	L	P		PCG	40
11.850	− 38	− 65	5	L	O		MOG	37
11.850	− 38	− 65	5	L	O	2 mm	MOG	19
11.850	− 38	− 65	5	L	O	3 mm	MTG	37
11.787	− 20	60	20	L	F		SFG	10
11.787	− 20	60	20	L	F	2 mm	MFG	10
11.665	38	33	14	R	F		IFG	46
11.665	38	33	14	R	F	2 mm	MFG	46
11.275	0.0	− 69	− 24		Cbl			
11.259	58	− 20	40	R	P		Post-C	3
11.259	58	− 20	40	R	P	3 mm	Pre-C	4
11.089	57	− 46	− 11	R	T		ITG	20
11.089	57	− 46	− 11	R	T	2 mm	MTG	37
(LC–CC) attentional cueing in acute hypoxia								
6.561	− 19	54	12	L	F		SFG	10
5.924	− 19	62	24	L	F		SFG	10
5.924	− 19	62	24	L	F	2 mm	MFG	9
4.277	19	52	− 5	R	F		ACC	10
4.277	19	52	− 5	R	F	3 mm	ACC	32
1.461	57	− 25	− 13	R	T		MTG	20
1.461	57	− 25	− 13	R	T	3 mm	ITG	21
2.135	19	− 71	− 37		Cbl			
(LCmot–CC) attentional cueing in ambient-air								
9.883	19	52	− 5	R	F		ACC	10
9.883	19	52	− 5	R	F	3 mm	ACC	32
9.676	− 19	54	12	L	F		SFG	10
8.654	19	− 37	61	R	P		Post-CG	3
8.654	19	− 37	61	R	P	2 mm	Post-CG	4
8.654	19	− 37	61	R	P	3 mm	STG	22
3.382	− 19	− 87	− 9	L	O		LG	18
3.382	− 19	− 87	− 9	L	O	3 mm	MOG	18
3.382	− 19	− 87	− 9	L	O	3 mm	FG	18
(LCmot–CC) attentional cueing in acute hypoxia								
13.974	− 19	54	12	L	F		SFG	10
13.700	− 19	− 60	44	L	P		Pre-Cun	7
13.700	− 19	− 60	44	L	P	2 mm	SPL	7
13.335	19	52	− 5	R	F		SFG	10
13.335	19	52	− 5	R	F	3 mm	ACC	32
13.335	19	52	− 5	R	F	3 mm	ACC	10
12.508	19	− 37	61	R	P		Post-C	5
12.418	19	− 71	− 37		Cbl			
12.313	− 38	− 65	5	L	O		MOG	37/19
12.313	− 38	− 65	5	L	O	3 mm	MTG	37/19

Note that active generators are ranked as a function of their magnitudes. Note also that different source locations are reported as a function of the region of interest (i. e., ROI), and that no ROI is reported where source location or Brodmann area (i. e., BA) did not change within a cube range of 3 mm, starting with 1 mm. Tailarach’s and Tournoux’s 3D coordinates (T) and ROIs are indicated in millimeters (mm).

Grid = 15 mm; Tikhonov correction SNR = 3.

*BA* brodmann area, *Cbl* cerebellum, *Hem* hemisphere, *L* left, *R* right, *F* frontal, *FG* fusiform gyrus, *IFG* inferior frontal gyrus, *ITG* inferior temporal gyrus, *LG* lingual gyrus, *Magn* magnitude, *Med* medial, *Mid* middle, *O* occipital, *MOG* middle occipital gyrus, *MTG* middle temporal gyrus, *P* parietal, *Post-C* post central gyrus, *Pre-C* precentral, *Pre-Cun* precuneus, *SFG* superior frontal gyrus, *SPL* superior parietal lobule, *STG* superior temporal gyrus.

**|**= Intermediate; – = No information and None BA, *ROI* Region of interest.

The results showed that, compared with ambient-air, ERPs elicited by phasic, informative cueing during acute hypoxia were associated with stronger activity in the right-sided anterior cingulate cortex (ACC, BA 32 and BA10) and in the left-sided superior frontal gyrus (SFG), middle and inferior temporal gyri of temporal cortex (MTC and ITC; BA 20 and 21, respectively), superior parietal lobule (SPL) and Precuneus (see again Fig. 11 for anatomical localization of these sources and Table 4 for spatial coordinates and specific features of the computed dipoles).

Most importantly, the activation of the right-sided ACC found for the lower requesting workload LC cueing condition, was appreciated with much greater magnitudes (i.e., 13.335) for the higher requesting workload LCmot cueing condition. This anterior strong activation was accompanied by a strong posterior activation of the left-sided superior parietal lobule (SPL, BA7) and/or precuneus (BA7), together with that of the middle occipital gyrus and/or of the middle temporal gyrus (MOG and MTG, BA 19/37 as well of MTG, BA37/19, respectively) as can be appreciated both in Fig. 11 and Table 4.

## Discussion

The main aim of the study was to investigate the effects of oxygen deficiency on the cued shifting of visual attention in space as mirrored in the cue-target time span by attention-orienting ERP deflections, namely the anterior ADAN/CNV and the parieto-occipital LDAP/TP, contralateral to the attention direction shift. Thanks to the use of a modified version of Attention Network Test^15^, we first replicated previous findings showing how ADAN/CNV and LDAP/TP are closely related to visuospatial orienting of attention^25,26,28–30,34,54,55^. In addition, our findings further support the view that ADAN/CNV reflect an integration of neurocognitive processes such as visuospatial selective processing, task preparation, and motor programming and execution.

However, going further ahead the present data also suggest that no matter respiratory condition and target-related location, these functional subdivisions seem to be closely related to a neuroanatomical segregation. Indeed, despite ERPs renown poor spatial resolution, at FC_3/4_ and F_3/4_ electrode sites only, much closer to brain motor and premotor areas, there were not any activation differences across the cueing mode/tasks, strongly suggesting the rising of a pre-target unspecific motor activation and preparation not directly related to the target-arrow pointing direction, and, in turn, to the specific instructed hand and finger to be used for responding to the target (Fig. 6).

Conversely, at prefrontal and fronto-polar electrode sites, namely, F_7/8_, AF_3/4_, and Fp_1/2_ scalp sites, a significant activation of attention orienting network, but not of alerting network, was observed, thus indicating that these brain regions were more closely related to visuospatial attentional processing^22,23,38^ rather than to action motor programming and execution. Most interestingly, the further finding of a lack of any diversities in ADAN/CNV amplitude between Air and Hypoxia conditions at FC_3/4_ and F_3/4_ electrode sites, in the light of the greater response amplitude of this component for the latter with respect to the former respiratory mode at the prefrontal and fronto-polar scalp regions (see Fig. 5 again), regardless of cueing mode and target-related location, not only further support the view of the aforementioned functional and neuroanatomical segregation of these neurocognitive functions, but also of more specific effects of hypoxia on visuospatial attention orienting.

Whilst the low spatial resolution of ERPs necessitates careful consideration when attempting anatomical localization, the scalp topographic distribution of brain potentials recorded within the 400–470 time window across the three attentional conditions largely validates the attentional control model postulated by^45^. This model posits that two partially segregated systems exist, each performing distinct attentional functions. According to this model, one system, involving the superior frontal cortex and intraparietal cortex, would be responsible for voluntary attention and selecting stimuli and responses. Another system, involving the temporo-parietal junction and inferior frontal cortex, specifically in the right hemisphere, would be specialized in detecting relevant stimuli that are salient or unexpected. This exogenous system contributes to the activation of prefrontal areas when sensory-perceptual processing identifies stimulus-driven cues to the validity of spatial location. The prefrontal areas subsequently provide feedback to the occipito-temporal-parietal regions, improving the processing of stimuli presented at that location. The discovery that this phenomenon occurs to a varying extent depending on the relevance of the location suggests that the gain of the visual signals may be adjusted in response to the relevance of stimuli and interference from distractors. In the present study, the whole-head topographic maps of brain voltages for both the phasic-alerting and spatially valid orienting cueing modes (i.e., LC and LCmot) in the air condition seem to reflect the activation of the exogenous system (see Fig. 3A and B).

Indeed, voltage maps point at a hemispheric asymmetry with a greater and more distributed occipito-parietal and temporal LDAP/TP over the right-sided electrode-sites with respect to a somehow more focused left-sided occipital-parietal and posterior temporal LDAP/TP for both these exogenous, spatially informative cueing modes. In support of a reflexive activation, as proposed by Corbetta's and Shulman's^45^ model, it is noteworthy that posteriorly centered trends for LDAP/TP are closely related to the absence of scalp-recorded ADAN/CNV over the anterior regions of the scalp. Unlike what is shown by the spatially uninformative CC mode and the utterly target-related exogenous NC cueing mode, which both display a prominent anterior ADAN/CNV and a lack of any posterior LDAP/TP.

To the extent that CNV represents inhibiting or suppressing ongoing neural and behavioral activities^22,23,35–37^, the significant negative potential distributed over the fronto-parietal regions of the scalp and the complete absence of posterior LDAP for the two spatially uninformative cueing modes/tasks (i.e. CC and NC) is likely to indicate a suppression of functional activation in posterior visual areas regarding target-related reflexive attention orienting, initiated by anteriorly-centered brain areas, probably due to varying levels of spatial information uncertainty from the two cueing modes.

Moreover, the rise in ADAN/CNV compound over the prefronto-polar and prefrontal regions of the scalp, together with that of posteriorly centred LDAP, for the spatially informative cueing modes/tasks (i.e. LC and LCmot) during acute hypoxia seems to indicate that the latter respiratory condition alters the right-sided exogenous attention shifting mechanisms. This requires an increased workload for both stimulus-driven visual processing and prefrontal control in order to receive sensory information and reflexively respond to the valid spatial cues.

This pattern is primarily evident in the hemifield related to targets above the horizontal meridian (refer to Fig. 3a) but is also present in the hemifield related to targets below the horizontal meridian, despite the noticeable variations in the polarity and distribution of the event-related potentials across the scalp (refer to Fig. 3b). In our opinion, the striking changes to CC-related ADAN/CNV voltage amplitude and scalp distribution, based on respiratory condition and target location, strongly support these views. Further to our research findings, it appears that there are distinct yet closely linked interrelationships between the activation of the anterior and posterior brain regions, as demonstrated by the ADAN/CNV and LDAP/TP components reflected in scalp-surface ERP slow potentials. This is because recent research into "brain interconnectivity theory"^49^ proposes a division of labor across cortical areas. Specifically, anterior executive control areas induce attention shifting in space and sustain it over time, while visual cortical areas are closely related to a corresponding biased preparation for later target processing.

Upon critical analysis of additional variables, including the modulation of alerting and orienting networks, as well as the delivery location of upper and lower targets in this study, we were able to demonstrate fascinating functional trends closely linking ADAN/CNV and LDAP/TP. As a whole, it was observed that when the magnitude of ADAN/CNV was low over frontal scalp areas, the posterior LDAP/TP increased in hypoxia compared to ambient air (refer to Fig. 3A and B for further details). This trend held for both the informative LC and LCmot spatial mode/tasks as well as for the uninformative alerting CC mode. Very interestingly, the fact that in hypoxia this alteration concerns CC mode too might be interpreted as results of a reduced rearward frontal cortical excitability, associated to focalized fronto-polar and dorsolateral prefrontal cortical excitability levels, which would, in turn, induce the rising of a LDAP/TP activity (Fig. 3A and B).

Importantly, ADAN/CNV data showed that this negative slow drift was largest for the cueing condition in which participants knew that they had to perform a target-related motor-response double choice according to the arrow-target type administered (i.e., LCmot) as compared to the single-choice cueing mode/task. This finding hints at an allocation of a further cognitive, besides motor, amount of processing resources during the anticipatory time span prior the target in LCmot cueing mode with respect the single-choice informative LC cueing mode. In our view, this finding is most important because the indicated largest ADAN/CNV for this higher work loaded LCmot condition is unrelated to any feature difference whatsoever in the cue stimulus, since the latter was identical to the one administered in the low workload informative spatial condition. It seems, then, that it was only subjects’ knowledge of a later more effortful target-type discrimination and motor response choice than in LC that induced them to allocate a greater amount of attentional processing resources.

The distribution and size of ADAN/CNV activity differs based on the target-related delivery location. Below-HM delivery location shows a larger and more distributed activity over fronto-polar and dorsolateral prefrontal sites, including frontal scalp sites. In contrast, above-HM delivery location shows more focused activity primarily on fronto-polar and prefrontal scalp regions.

This pattern of results was true not only for the spatially informative cueing mode/tasks (i. e., LC and LCmot), but for the alerting but spatially uninformative CC cueing mode too, despite at a much reduced extent. Based on these trends, we believe that our findings provide robust support for the proposed view that the anterior ADAN/CNV recorded at dorsolateral prefrontal sites and frontal electrode sites is the expression of suppression processes of ongoing activity for preparation and orientation during the time between warning and target in view of performing a required rapid response to the task^22,23,35–38^. This was reflected at the scalp by the marked reduction of LDAP/TP component amplitude at posterior brain visual scalp areas during cue and target preparation biasing.

The data showed that there are significant changes in the brain's functional activation patterns during the cue-target interval in hypoxia compared to ambient air. These changes are more pronounced in hypoxia thus suggesting that acute normobaric hypoxia affects visuospatial attention shifting in space. The fact that these alterations also affect the CC mode suggests a decrease in frontal cortical excitability, particularly in the fronto-polar and dorsolateral prefrontal areas. This decrease in excitability may lead to increased activity in the LDAP/TP regions at a lower level. This finding fits well with previous reports of an attentional anisotropy (e.g., Refs.^46–48^). Interestingly, further findings indicated that these visual field horizontal meridian-centered effects held only for the spatial attention informative modes, namely LC and LCmot ones. These findings suggest that the anisotropy manifests only for spatial attention orienting and action preparation processing (e.g., Refs.^46–48^. Indeed, ADAN/CNV was larger for LCmot than for LC and CC, and, in turn, for LC than for CC, thus hinting at a greater neurocognitive workload for the former cueing mode. To our knowledge, this seems to be the first evidence of the prevalence of the psychomotor component of ADAN/CNV in the ANT test. At this regard, it is interesting to consider the left-hemispheric asymmetry of the prefrontal ADAN/CNV only for the valid LC and LCmot spatial attentional shifting found in ambient air condition with respect to hypoxia (see Fig. 7 again; see also citation of a left hemisphere role in motor planning advanced by Posner^22^ in “Introduction” section).

At this regard, for comparability’s sake with previous ANT-based studies and for a check of data robustness, we verified that our LDAP/TP findings in ambient air are rather consistent with Neuhaus’s et al.^55^ modulation pattern of cue-related activation for CC and LC modes at posterior electrodes, namely O_1_-O_2_, PO_9_-PO_10_, P_z_, and P_4_, developing just before target delivery. As far as ADAN/CNV is concerned, despite Neuhaus et al.^55^ did not provide any measures about this component, the waveform trend for NC, as visible in their paper figures (e.g., Figs. 3 and 4), was also consistent with our own and Mento’s^56^ findings of a noticeable negative drift during the cue-target time span, no matter the absence of any prior cue presentation. This finding may reflect participants' sustained allocation of attentional resources over time or a tonic state of alertness or vigilance to manage this ANT subtask.

In our view it is relevant that our ADAN/CNV results are also consistent with Galvao-Cardona’s et al.^44^ findings of a larger attentional modulation of ‘orienting’ but not ‘alerting’ network, in the light of our original ANT-based time span of 500 ms, with respect to the prolonged cue-target time of 1 s used by these authors for boosting CNV rising and measurement. On the other hand, the present ADAN/CNV findings are, instead, not consistent with those of Abundis-Gutierrez et al.^42^ and Luna et al.^43^ who, using a short 600 ms visual cue-target ISI with the addition of an alerting auditory tone cue and the introduction of invalid, besides valid, trials, reported that, unlike attention orienting, only phasic alerting induced a significant CNV modulation at anterior scalp sites. We believe that it might be reasonably thought that these controversial findings derived from the introduction of the indicated further task attributes by the quoted studies.

The present results seem to indicate that ERP amplitudes were robustly modulated by top-down processes, i.e., the amount of cue-related spatial information and preparation processes allocated to direct visuospatial attention. Lowest amplitudes related to the condition without any preceding cue (NC); intermediate amplitudes related to CC alerting condition; and largest amplitudes related to the two LC and LCmot spatial cueing conditions, with the latter eliciting a larger pre-target activation than the former.

Since our findings were not as a whole completely in agreement with Woldorff’s et al.^31^ and Grent’-T-Jong’s et al.^32^ findings of a *Biasing-Related Negativity* (BRN), we attributed these differences to the discrepant paradigms used in various studies on BRN. For example, the use of a central cueing mode, a long cue-target ISI, and the comparison of a valid spatial cueing instructing to shift attention to a point in space with a “to be interpreted” cueing, randomly instructing to refrain from shifting attention as opposed to a peripheral cueing mode, a short cue-target ISI, and the contrast between a spatially informative valid cueing condition indicating to shift attention to a point in space and a spatially uninformative, but alerting, condition, and/or an utterly lacking any cue one, in our own paradigm. Also, in the view of the more recent and articulated “brain interconnectivity theory” advanced by Snyder et al*.*^49^, the central-cueing-related BRN may possibly reflect a modulation of the processing activation of the visual brain areas deriving from the top-down influences from higher-order pre-frontal brain regions for facilitating or suppressing target processing and discrimination in spatially informative and uninformative cueing modes for attention-shifting, respectively. Conversely, at least for the above-HM visual field in air, the positive LDAP/TP developing in our peripheral cueing paradigm seems to mirror a bottom-up or reflexive activation to salient, spatially informative stimuli somehow disengaged from the frontal system influence.

Since the raise of CNV was measured from 100 ms up until 30 ms prior target presentation as a function of cueing/task conditions, our findings of significant differences across cueing/tasks strongly support the view that this component does not simply reflect an aspecific dorsolateral prefrontal and frontal activation related to alerting or general preparation, but also to a spatial orienting of attention in response to the spatially informative cues, finalized to the optimization of the processing of the following target by the posterior, occipito-temporal brain areas. Conversely, within the same prior target latency range, the NC condition showed an apparently curtailed, albeit sustained over time raise of negativity.

Overall, hypoxia triggered a greater frontal slow negative shift for the phasic (i.e., CC) but not for the tonic (i.e., NC) alerting, besides for the orienting of attention-based double-choice task, but not for the single-choice task.

These findings suggest that at least at non-lethal levels of hypoxia at which volunteers were exposed in our study (i.e., 12.5% oxygen saturation), this oxygen deficiency did not alter cue-related brain attention shifting control mechanisms and visual cortical areas priming, but as a function of the valid spatially centered information and task workload coped with. Indeed, alterations of brain attention shifting control in hypoxic conditions were observed only for the two cueing conditions possibly characterized by greater amount of spatial information available, namely, the single button-press LC condition and the double-choice, button-press-related, LCmot phasic alerting and spatially informative conditions. As for the latter condition, quite certainly it was characterized by higher levels of cognitive and psychomotor preparation workload. At this regard, it is possible that, despite a generalized impact of hypoxia on brain physiology and functions, the lower workload proper of the single-choice button press, phasic-alerting, and spatially informative condition (i.e., LC) could be dealt allocating lower amount of processing resources, as reflected by lower levels of ADAN/CNV found in hypoxia at more anterior fronto-polar and dorsolateral prefrontal electrodes (i. e., F_7/8_, AF_3/4_ and PF_1/2_), and, in turn, of LDAP/TP at posterior scalp regions.

Another important issue to be considered concerns our search for separate, but close, interrelationships between brain anterior and posterior districts, as theorized by several authors (e.g. Refs.^31,32,49^). The present findings not only provided further evidence of these interrelationships, but also of a neurofunctional organization according to which (unlike for CC and NC modes), an increase of ADAN/CNV over anterior scalp regions for LC and LCmot cueing modes, corresponded to a pronounced decrease of LDAP/TP at posterior visual scalp regions. These electro-functional activation patterns are closely related to the target-related delivery location, in that, when ADAN/CNV is larger for below-HM target-related location, with respect to above-HM target-related location, LDAP/TP shows a vice versa trend. Namely, it is larger for the above-HM than the below-HM target-related delivery location.

In addition, regardless of site and hemisphere, hypoxia markedly increased ADAN/CNV, but decreased LDAP/TP, respectively, for both target-related presentation positions for both the LC and LCmot attention-shifting modes. Due to these changes, while in hypoxia ADAN/CNV and LDAP/TP revealed to be of higher and lower amplitude, respectively, than in ambient air for both target-related delivery locations, where LC attention orienting mode was concerned, for LCmot this pattern was true for the above-HM target delivery location condition only. Notwithstanding the larger amplitude of LDAP/TP obtained in hypoxia than in ambient air for CC mode, the indicated functional activation patterns apparently do not apply consistently neither to alerting CC mode nor to the warning absent-related NC cueing mode.

As for the behavioral data, our findings indicated that valid attention orienting speeded up overt motor response, regardless of target congruency. These results are closely consistent with previous findings (e.g., Refs.^47,48^), and further support the view of an anisotropic attentional control of visual space, according to which upper visual field is characterized by a higher perceptual detection and discrimination sensitivity as well as response speed than the lower visual field, originally advanced by Ref.^46^.

All in all, our findings seem to imply that information gained by means of pre-target preparatory processes is strictly reflected by speed of overt target-related motor responses, notwithstanding the additional information derived from target processing stages preceding these overt responses. The findings of this study suggest that both the location at which a target is presented, and the presence of hypoxia negatively affect the speed of motor responses. When the target was presented above the visual field meridian, response times were faster compared to when it was presented below the visual field meridian. However, hypoxia had a more significant negative impact on visuospatial attention compared to target location. In the air session, attention orienting modes induced faster response times compared to hypoxia, but acute oxygen shortage impaired attention alerting and orienting modulation.

In conclusion, the present findings provided clear evidence that acute normobaric hypoxia has marked detrimental effects on both overt motor performance and electrophysiological activations undergirding the shifting of attention in the visual space, as reflected by scalp-recorded ADAN and LDAP components. These effects are characterized by an inverted trend at anterior and posterior scalp regions, in that, whenever anteriorly recorded ADAN/CNV shows a prominent magnitude at fronto-polar and dorsolateral prefrontal scalp regions for informative cueing modes, the posterior LDAP/TP amplitude is markedly decreased. Furthermore, this inverted trend shows remarkable differences between the above-HM and lower-HM regions of visual space. Indeed, whenever ADAN/CNV is hardly detected for above-HM, LDAP/TP shows a prominent amplitude very likely due to automatic reflexive mechanisms. Conversely, an increase of ADAN/CNV at anterior fronto-polar and dorsolateral prefrontal scalp regions for below-HM visual space remarkably suppressed the LDAP/TP height. This pattern occurred at a greater extent in hypoxia with respect with ambient air, thus indicating the detrimental effects of oxygen deficiency on attention mechanisms.

To the extent that ADAN/CNV represents a process of suppression of information processing, the absence of it as in ambient air or a moderate amplitude of it as in acute hypoxia, as found in the case of the superior visual hemifield for both the LC and LCmot conditions, seems to be related to a clear posterior activation, indexed by an increasing amplitude of LDAP/TP.

Source reconstruction computed on the difference waves between ERP responses to both LC and LCmot spatial informative conditions and CC condition separately for ambient air and acute hypoxia also indicated how the latter respiratory mode induced the activation of anterior right-sided ACC besides of left-sided SFG, SPL and Precuneus for sustaining additional processing resources and control. LORETA source reconstruction also showed enhanced posterior activation during hypoxic conditions, and this agrees with available literature suggesting that normobaric hypoxic exposure does not affect early visual processing and posterior metabolism (Blacker and McHail^12^). It is possible that the ACC-related increase of ADAN/CNV might reflect a compensatory mechanism from executive control systems to cope with the reduction of oxygen in the air.

## Data Availability

The data that support the findings of this study are available from the corresponding author, upon reasonable request.
